# Model-based and Model-free Machine Learning Techniques for Diagnostic Prediction and Classification of Clinical Outcomes in Parkinson’s Disease

**DOI:** 10.1038/s41598-018-24783-4

**Published:** 2018-05-08

**Authors:** Chao Gao, Hanbo Sun, Tuo Wang, Ming Tang, Nicolaas I. Bohnen, Martijn L. T. M. Müller, Talia Herman, Nir Giladi, Alexandr Kalinin, Cathie Spino, William Dauer, Jeffrey M. Hausdorff, Ivo D. Dinov

**Affiliations:** 1 Statistics Online Computational Resource, Department of Health Behavior and Biological Sciences, University of Michigan, Ann Arbor, MI United States; 2 Department of Biostatistics, University of Michigan, Ann Arbor, MI United States; 3 Department of Statistics, University of Michigan, Ann Arbor, MI United States; 4 Department of Radiology, University of Michigan, Ann Arbor, MI United States; 5 Department of Neurology and Ann Arbor VA Medical Center, University of Michigan, Ann Arbor, MI United States; 6 Morris K. Udall Center of Excellence for Parkinson’s Disease Research, University of Michigan, Ann Arbor, MI United States; 7 The Center for the Study of Movement, Cognition and Mobility, Neurological Institute, Tel Aviv Sourasky Medical Center, Tel Aviv, Israel; 8 Sagol School of Neuroscience and Department of Physical Therapy, Sackler Faculty of Medicine, Tel Aviv University, Tel Aviv, Israel; 9 Department of Neurology and Sieratzki Chair in Neurology, Sackler School of Medicine, Tel Aviv University, Tel Aviv, Israel; 10 Rush Alzheimer’s Disease Center & Orthopaedic Surgery, Rush University, Chicago, IL USA; 11 Department of Computational Medicine and Bioinformatics, University of Michigan, Ann Arbor, MI 48109 USA; 12 Michigan Institute for Data Science, University of Michigan, Ann Arbor, MI 48109 USA

## Abstract

In this study, we apply a multidisciplinary approach to investigate falls in PD patients using clinical, demographic and neuroimaging data from two independent initiatives (University of Michigan and Tel Aviv Sourasky Medical Center). Using machine learning techniques, we construct predictive models to discriminate fallers and non-fallers. Through controlled feature selection, we identified the most salient predictors of patient falls including gait speed, Hoehn and Yahr stage, postural instability and gait difficulty-related measurements. The model-based and model-free analytical methods we employed included logistic regression, random forests, support vector machines, and XGboost. The reliability of the forecasts was assessed by internal statistical (5-fold) cross validation as well as by external out-of-bag validation. Four specific challenges were addressed in the study: Challenge 1, develop a protocol for harmonizing and aggregating complex, multisource, and multi-site Parkinson’s disease data; Challenge 2, identify salient predictive features associated with specific clinical traits, e.g., patient falls; Challenge 3, forecast patient falls and evaluate the classification performance; and Challenge 4, predict tremor dominance (TD) vs. posture instability and gait difficulty (PIGD). Our findings suggest that, compared to other approaches, model-free machine learning based techniques provide a more reliable clinical outcome forecasting of falls in Parkinson’s patients, for example, with a classification accuracy of about 70–80%.

## Introduction

### PD clinical characteristics, current state-of-the-art techniques, societal impact

Parkinson’s disease (PD) is a common neurodegenerative disorder that affects over 10 million people worldwide. PD affects about 1% of people over 60 years of age and the prevalence increases with age. People with PD experience a range of motor and non-motor symptoms that include tremor, rigidity, bradykinesia, postural instability, gait disturbances such as freezing of gait (FoG), autonomic disturbances, affective disorders, sleep disturbances, and cognitive deficits^1^. These symptoms markedly impact and curtail health related quality of life^2^. Freezing of gait and associated falls represent one of the most serious consequences of PD^3^. Falls are much more common in patients with PD than in age-matched controls and falls often lead to reduced functional independence, increased morbidity, and higher mortality^4^. The ability to better identify future fallers from non-fallers could inform more effective treatment and personalized medicine planning.

The hallmark pathology of PD is loss of dopamine in the striatum secondary to progressive degeneration of dopaminergic cells in the substantia nigra pars compacta, accompanied by the formation of Lewy bodies^5^. A variable combination of tremor, rigidity, and bradykinesia symptoms may present along with postural instability and gait difficulty (PIGD) features. Because of primary involvement of the basal ganglia in PD, it has often been asserted that these motor features are mainly attributable to nigrostriatal dopaminergic loss. A common dopamine replacement therapy to ameliorate PD motor symptom is levodopa (L-DOPA). A recent study from Vu *et al*.^6^ showed that L-DOPA potency was lowest for PIGD features compared to other cardinal motor features. In the Sydney Multicenter Study of PD, patients have been followed for about two decades. Results of this study indicate that dopamine non-responsive problems dominate 15 years after initial assessments and include frequent falls, which occurs in 81% of the patients^7^. Similar findings were recently reported by López *et al*. after following de novo PD patients for 10 years^8^. These authors reported good responses to dopaminergic treatment in the first year with a progressive decline, becoming more manifest especially after 3 years. Significant PIGD motor disabilities arose at 10 years in 71% of patients that were mainly caused by non-dopamine-responsive features such as freezing of gait (FoG)^8^. The L-DOPA resistance of PIGD motor features has been proposed to include non-dopaminergic structures in widespread brain regions^9^. As axial motor impairments, in particular falls, do not respond well to dopaminergic medications there is a need to identify early predictors of falls. Such predictors may provide potential clues about underlying mechanism of falls that may more effectively inform future treatment interventions. The main goal of this study was to identify clinical and MR imaging predictors of falls from two independent archives containing clinical and imaging data of PD patients.

### Machine Learning methods for prediction, classification, forecasting and data-mining

Both model-based and model-free techniques may be employed for prediction of specific clinical outcomes or diagnostic phenotypes. The application of model-based approaches heavily depends on the a priori statistical statements, such as specification of relationship between variables (e.g. independence) and the model-specific assumptions regarding the process probability distributions (e.g., the outcome variable may be required to be binomial). Examples of model-based methods include generalized linear models. Logistic regression is one of the most commonly used model-based tools, which is applicable when the outcome variables are measured on a binary scale (e.g., success/failure) and follow Bernoulli distribution^10^. Hence, the classification process can be carried out based on the estimated probabilities. Investigators have to carefully examine and confirm the model assumptions and choose appropriate link functions. Since the statistical assumptions do not always hold in real life problems, especially for big incongruent data, the model-based methods may not be applicable or may generate biased results.

In contrast, model-free methods adapt to the intrinsic data characteristics without the use of any a priori models and with fewer assumptions. Given complicated information, model-free techniques are able to construct non-parametric representations, which may also be referred as (non-parametric) models, using machine learning algorithms or ensembles of multiple base learners without simplification of the problem. In the present study, several model-free methods are utilized, e.g., Random Forest^11^, AdaBoost^12^, XGBoost^13^, Support Vector Machines^14^, Neural Network^15^, and SuperLearner^16^. These algorithms benefit from constant learning, or retraining, as they do not guarantee optimized classification/regression results. However, when trained, maintained and reinforced properly and effectively, model-free machine learning methods have great potential in solving real-world problems (prediction and data-mining). The morphometric biomarkers that were identified and reported here may be useful for clinical decision support and assist with diagnosis and monitoring of Parkinson’s disease.

There are prior reports of using model-free machine-learning techniques to diagnose Parkinson’s disease. For instance, Abos *et al*. explored connection-wise patterns of functional connectivity to discriminate PD patients according to their cognitive status^17^. They reported an accuracy of 80.0% for classifying a validation sample independent of the training dataset. Dinesh and colleagues employed (boosted) decision trees to forecast PD. Their approach was based on analyzing variations in voice patterns of PD patients and unaffected subjects and reported average prediction accuracy of 91–95%^18^. Peng *et al*. used machine learning method for detection of morphometric biomarkers in Parkinson’s disease^19^. Their multi-kernel support vector machine classifier performed well with average accuracy = 86%, specificity = 88%, and sensitivity = 88%. Another group of researchers developed a novel feature selection technique to predict PD based on multi-modal neuroimaging data and using support vector classification^20^. Their cross-validation results of predicting three types of patients, normal controls, subjects without evidence of dopaminergic denervation (SWEDDs), and PD patients reported classification accuracy about 89–90%. Bernad-Elazari *et al*. applied a machine learning approach to distinguish between subjects with and without PD. Their objective characterization of daily living transitions in patients with PD used a single body-fixed sensor, successfully distinguishing mild patients from healthy older adults with an accuracy of 86%^21^. Previously identified biomarkers, as well as the salient features determined in our study, may be useful for improving the diagnosis, prognosticating the course, and tracking the progression of the disease over time.

### Study Goals

This study aims to address four complementary challenges. To address the need for effective data management and reliable data accumulation, *Challenge 1* involves designing a protocol for harmonizing and aggregating complex, multisource, and multi-site Parkinson’s disease data. We applied machine learning techniques and controlled variable selection, e.g., knockoff filtering^22^, to address *Challenge 2*, identify salient predictive features associated with specific clinical traits, e.g., patient falls. *Challenge 3* involves forecasting patient falls using alternative techniques based on the selected features and evaluating the classification performance using internal (statistical) and external (prospective data) validation. Finally, *Challenge 4*, addresses the need to forecast other clinically relevant traits like Parkinson’s phenotypes, e.g., tremor dominance (TD) vs. posture instability and gait difficulty (PIGD)^23^.

### Predictive Analytic Strategy

**Figure 1:**
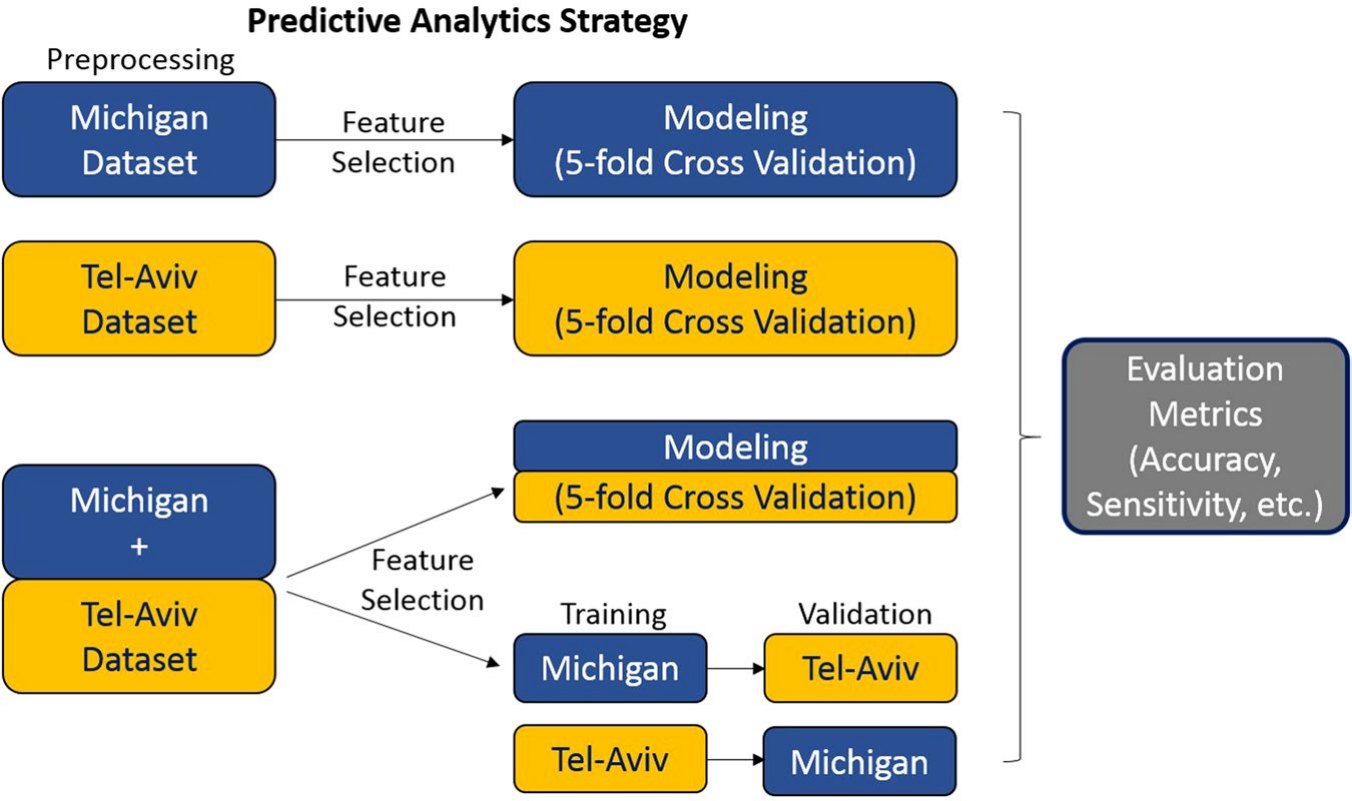
Predictive Analytics Strategy: (Top) Identify critical features and build predictive models independently on the Michigan and the Tel-Aviv datasets, respectively. (Bottom) Harmonize and merge the two data archives and perform the same analytics on the aggregate data. The bottom-right branch of the diagram illustrates the process of training the models on one of the datasets and (externally) validating their accuracy on the other complementary dataset.

The datasets used in this study were collected independently at two sites – the University of Michigan Udall Center of Excellence in Parkinson’s Disease Research (Michigan data) and the Sourasky Medical Center, Israel (Tel-Aviv data). Both the datasets include high dimensional data consisting of several hundred demographic and clinical features for about a couple of hundred PD patients. This research is focused primarily on the prediction of patients’ falls, although alternative clinical outcomes and diagnostic phenotypes can be explored using the same approach. As not all of the features in the clinical record are strongly associated with each specific response, our goal is to identify some important critical features, build the simplest statistical models, and demonstrate reproducible computational classifies that produce higher prediction accuracy while avoiding overfitting. Figure 1 shows a high-level schematic of the study-design, including the complementary training and testing strategies.

In general, model-free statistical learning methods (e.g. Random Forest, Support Vector Machines) make fewer assumptions and often outperform model-based statistical techniques like logistic regression, which is often considered a baseline method, on large and complex biomedical data^24–27^. To quantify the forecasting results, we used established evaluation metrics such as overall accuracy, sensitivity, specificity, positive and negative predictive power, and log odds ratio. For clinical datasets with a large number of features, it is difficult to avoid the multi-collinearity problem, which causes problems with maximum likelihood estimation of model-based techniques^28^. As the machine learning techniques have minimal statistical assumptions, they may provide more flexible and reliable predictions.

This manuscript is organized as follows: The methods section describes the study design, the characteristics of the data and meta-data, the preprocessing, harmonization, aggregation and analysis methods, as well as the evaluation strategies. The results section reports the findings for each of the study designs shown in Fig. 1. Finally, the discussion section explains the findings, identifies potential drawbacks and suggests prospective translational studies.

## Methods

All methods and analyses reported in the manuscript were carried out in accordance with relevant institutional, state and government guidelines and regulations. The experimental protocols were approved by the institutional review boards of the University of Michigan (HUM00022832) and Tel Aviv Sourasky Medical Center (0595–09TLV). Informed consent was obtained from all participating volunteers prior to enrollment in the study and data collection.

### Data sources and management

Below we describe the two main sources of data (University of Michigan and Tel Aviv Sourasky Medical Center) and discuss the data management, wrangling, preprocessing, imputation, harmonization, aggregation, and analytics.

#### Michigan data

The University of Michigan archive included data collected as part of a NIH-funded clinical and neuroimaging study of PD. Additional information about inclusion/exclusion criteria and data dictionary are provided in Supplementary Materials Section I.1.a. Briefly, the raw dataset compiled at Michigan contains study subjects’ demographics, PET, behavioral and sensory assessments, Mattis Dementia Rating Scale, sleep questionnaires, genetics, number of falls, clinical measures and MR neuroimaging (207 variables in total). Among the 225 study subjects, there were 148 patients with Parkinson’s disease and 77 healthy participants.

#### Tel-Aviv data

The Tel-Aviv archive includes demographic, clinical, gait, balance and imaging data. The dataset was originally gathered to study the role of white matter changes in PD and putative relationships to motor phenotypes^29,30^. The study included 110 patients with idiopathic PD recruited by referrals from specialists at the outpatient movement disorders unit, and from other affiliated clinics. Additional information about inclusion/exclusion criteria and data dictionary are provided in Supplementary Materials Section I.1.b.

#### Michigan + TelAviv Data Aggregation

The preprocessed Tel-Aviv and Michigan datasets are harmonized and merged using 133 shared variables, which include Subject ID, PD subtype (TD vs. PIGD), Tremor score, PIGD score, gender, age, weight, height, BMI, Geriatric Depression Scale (short form), the Timed up and go test, specific items from Part I, II and III of the Movement Disorder Society (MDS)-sponsored version of the UPDRS, Hoehn and Yahr scale, Montreal Cognitive Assessment (MoCA), and 56 derived neuroimaging features. Notably, the UPDRS Part III sub items from the two datasets were both measured under the “OFF” medication cycle, i.e., approximately 12 hours of antiparkinsonian medication withdrawal prior to the assessments. The aggregated dataset consists of 251 subjects and 133 variables.

### Model-based and Model-free machine learning methods

The Supplementary Materials Section I.2 (Predictive Analytics) includes the mathematical descriptions of the model-based (e.g., Logistic Regression) and model-free (e.g., Random Forest, Adaptive and gradient boosting, Support Vector Machines, Neural networks, SuperLearner) techniques used for prediction and classification. The Knockoff filtering and random-forest feature selection methods are detailed in Supplementary Materials Section I.3 (Feature Selection).

### Statistical validation strategies and evaluation metrics

#### Classification

To validate the prediction performance for binary classes, we usually construct a 2 × 2 contingency table (confusion matrix) as illustrated on Table 1:

**Table 1:** The confusion matrix provides a mechanism to assess the accuracy of binary diagnostic classification.

		Reference	
		Fall	Non-fall
Prediction	Fall	TP	FP
	Non-fall	FN	TN

**True Positive(TP)**: Number of observations that correctly classified as “Fall” group.

**True Negative(TN)**: Number of observations that correctly classified as “Non-Fall” group.

**False Positive(FP)**: Number of observations that incorrectly classified as “Fall” group.

**False Negative(FN)**: Number of observations that incorrectly classified as “Non-Fall” group.

**Accuracy(ACC)**: ACC = (TF + TN)/Total number of observations.

**Sensitivity (SENS) & specificity (SPEC)**: Sensitivity measures the proportion of “Falls” that are correctly classified while specificity measures the proportion of “Non-fall” that are correctly identified:

1 $$SENS=\,\frac{TP}{TP+FN},\,SPEC=\frac{TN}{TN+FP}.$$

**Positive Predictive Value (PPV) & Negative Predictive Value (NPV)**: Positive Predicted Value measures the proportion of true “Fall” observations among predicted “Fall” observations. Similarly, Negative Predicted Value measures the proportion of true “Non-fall” observations among predicted “Non-fall” observations:

2 $$PPV=\frac{TP}{TP+FP},\,NPV=\frac{TN}{TN+FN}.$$

**ROC Curve & Area Under the Curve (AUC)**: The Receiver Operating Characteristic (ROC) curve explicates the relation between true positive rate (i.e., sensitivity) and false positive rate (i.e. 100%-specificity) for various cut-offs of a continuous diagnostic test^31^. The performance of the test may be summarized by the aggregate area under the ROC curve (AUC); $0\le AUC\le 1$ and higher AUC indicates better performance. In this study, 5-fold cross validation is applied, the AUC is calculated for each repeated iteration, and the average AUC is reported as an overall quantitative estimate of classification performance, which can be used to compare alternative classifiers^32^.

### Statistical tests

A number of critical features from Michigan/Tel-Aviv/Combined datasets were identified during feature selection. As observed in density plots, data of clinical measurements were not normally distributed within sub-patient groups, hence two-sample t-test cannot be used. When comparing two independent samples (fall and non-fall patient group), non-parametric tests are implemented as they have the advantage of making no assumption about data distribution.

#### Mann-Whitney-Wilcoxon (MWW) test

Frequently treated as the non-parametric equivalent of the two-sample t-test, the MWW test is used to determine whether two independent samples from populations having the same distributions with the same median without assuming normal distributions^33^. The calculation is based on the order of the observation in samples. In this study, we used R-based wilcox.test() to carry out two-sided hypothesis testing procedure:

H_0_: The distributions of two samples do not differ by a location shift.

H_1_: The distribution of one population is shifted to the left or right of the other.

MWW test statistic: $U=W-\,\frac{{n}_{2}({n}_{2}+1)}{2}$, where $W$ is the rank sum statistic of one group and ${n}_{2}$ is the number of observations in the other group whose ranks were not summed. The $U$ statistic is reported and labeled as $W$^34^.

#### Kolmogorov–Smirnov (KS) test

Named after Andrey Kolmogorov and Nikolai Smirnov, it is one of the most useful and general non-parametric method that determines whether two independent samples differ significantly in both location and shape of the one-dimensional probability distributions. KS test^35^ quantifies the distance between the empirical distribution functions of two sample:

H_0_: The samples are drawn from the same distribution.

H_1_: The samples are not drawn from the same distribution.

The empirical distribution function: ${F}_{n}(x)=\frac{1}{n}\sum _{i=1}^{n}\,{I}_{[-\infty ,x]}\,({X}_{i})$, where n is the number of observations. Then, the KS test statistic is:

3 $${D}_{n,m}=\mathop{sup}\limits_{x}|{F}_{1,n}(x)-\,{F}_{2,m}(x)|,$$

where ${F}_{1,n}(x)$ and ${F}_{2,m}(x)$ are the empirical distribution functions of the first and second sample.

## Results

### Overall Summaries

**Figure 2:**
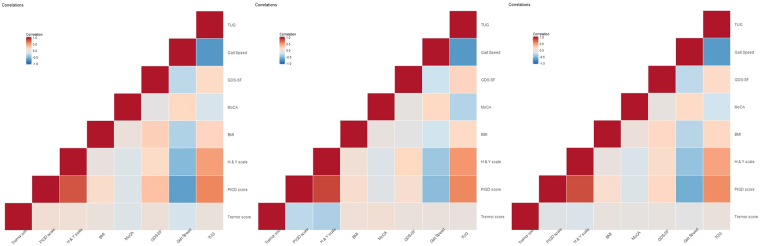
Pair correlations of some features, separately for each of the three datasets used in the study. (**A**) Michigan data boxplots illustrating significant differences in MDS_TREM (p = 0.5465), MDS_PIGD (p < 0.001), H and Y scale (p < 0.001), gaitSpeed_Off (p < 0.001) between PD patients with and without a history of falls, based on MWW test. (No = 0, Yes = 1). (**B**) Tel-Aviv data boxplots illustrating significant differences in Tremor_score (p = 0.01094), PIGD_score (p < 0.001), H and Y scale (p < 0.001) and FOG_Q (p < 0.001) between PD patients with and without a history of falls, based on MWW test. (No = 0, Yes = 1).

Table 2 shows the basic summary statistics for the three datasets and Fig. 2 illustrates correlation heatmaps of some core data features. There are some differences between the paired correlations between features and across data archives. For instance, gait-speed is strongly negatively correlated with tremor score, PIGD score, BMI, Hoehn and Yahr scale (H&Y), and GDS-SF (Geriatric Depression Scale - short form), whereas PIGD (MDS_PIGD) is strongly-positively correlated with TUG (Timed Up and Go test), GDS-SF, BMI, and Hoehn and Yahr scale. We also found that gait speed is negatively correlated with postural stability (pos_stab). The presence of more severe postural instability and gait difficulties is not robustly correlated with the non-motor experiences of daily living in the patient. The non-motor experiences of daily living reflect impairments of cognition, mood, sleep and autonomic functions. Although axial impairments are generally associated with cognitive impairments in PD, the lack of significant associations with overall non-motor experiences of daily living may be due to the heterogeneous (cognitive and non-cognitive) nature of this MDS UPDRS subscale.

**Table 2:** A summary table, with selected feature pair correlations, separately for each of the three datasets used in the study.

Cohort	Original Size(n)	Effective Size(m)	#Features*
Michigan	225(48)	148**(45)	179
Tel-Aviv	105(41)	103(41)	165
Aggregated	330(89)	251(86)	129

The values in parentheses represent the numbers of patients that had falls.

*Number of features after preprocessing.

**77 healthy controls were excluded.

#### EDA Plots for Michigan and Tel-Aviv Data

**Figure 3:**
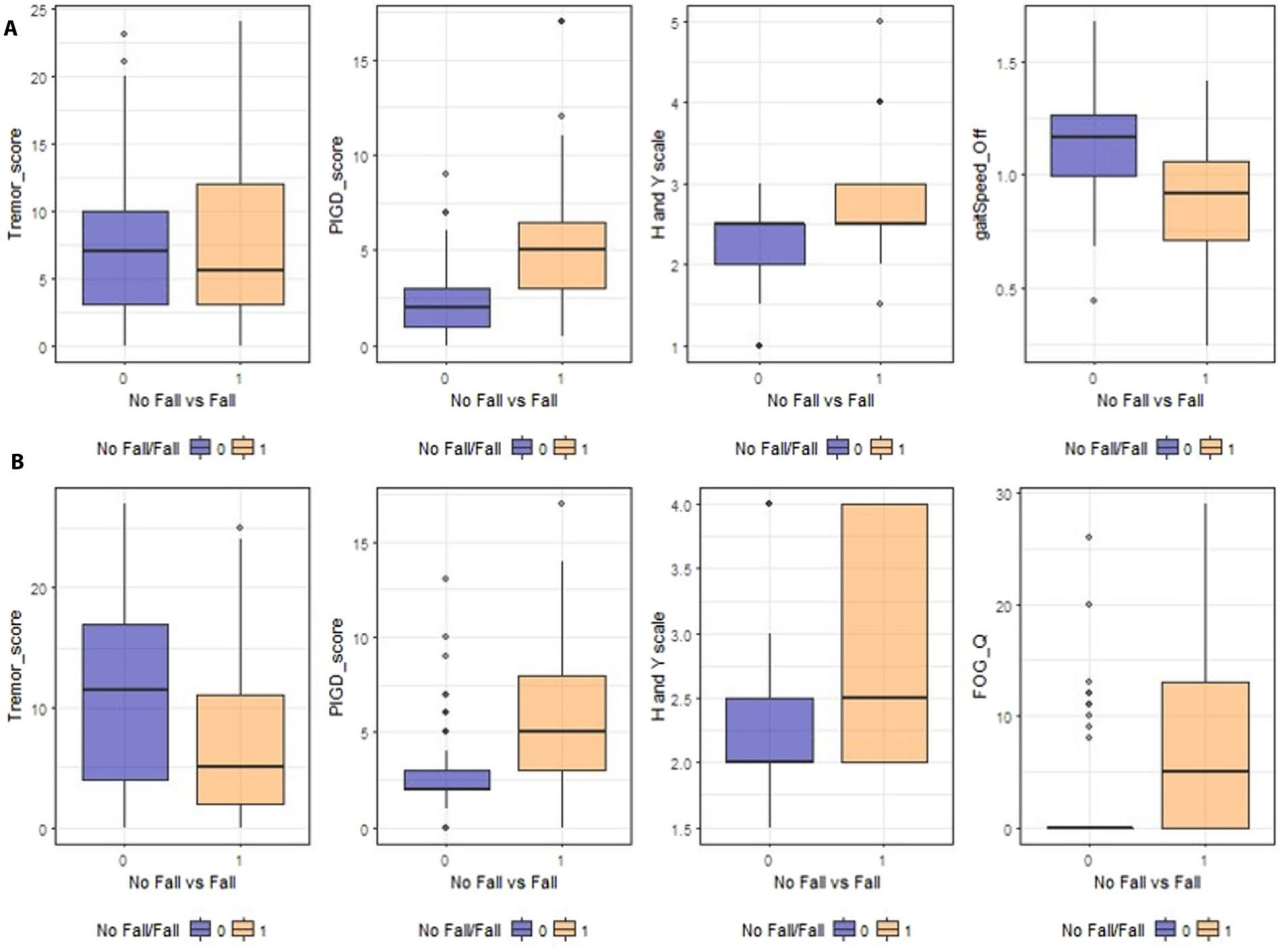
Exploratory data analytics illustrating some of the relations between falling and several clinical measures for the Michigan dataset (**A**) and the Tel-Aviv dataset (**B**), separately.

Figure 3 demonstrates exploratory data analytics (EDA) including univariate and multivariate distributions contrasting the Michigan and Tel-Aviv populations, also see Supplementary Figures S.3 and S.4.

### Missing Data Plots

**Figure 4:**
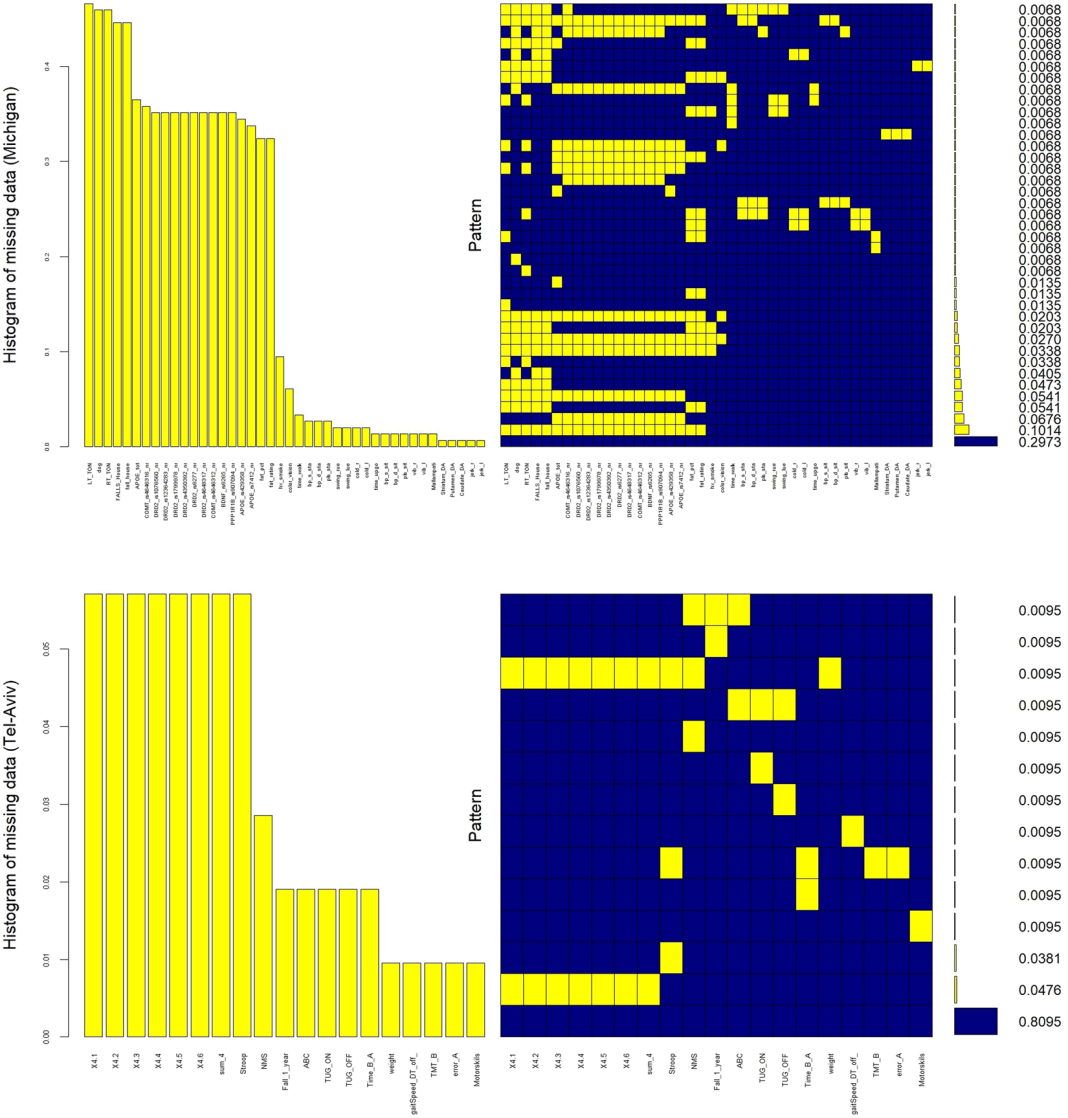
Missing patterns of Michigan (top) and Tel-Aviv (bottom) datasets. Approximately 30% of the Michigan study subjects have complete information, e.g., many cases have unrecorded genetic biomarkers. Data completeness is higher in Tel-Aviv data, missingness only occurred in about 19% of the participants.

Figure 4 illustrates the missing data patterns for both, the Michigan and the Tel-Aviv datasets. This lower dimensional projection suggests that the two cohorts are quite entangled, which may present a challenge in classification of falls/no-fall.

#### *Challenge 1*. Harmonizing and aggregating complex multi-source and multisite Parkinson’s disease data

**Figure 5:**
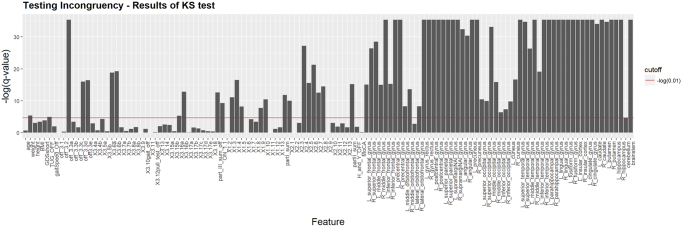
Results of KS tests on 126 features comparing the distributions in Michigan and Tel-Aviv data. The red horizontal line represents the cutoff of −*log*(*α*), where *α* (desired FDR) = 0.01.

Data Aggregation: Since the data were acquired in independent studies at two separate institutions, not all the features collected were homologous. Even common features contained in both archives had some with substantially different distributions, according to Kolmogorov–Smirnov test, Fig. 5.

Figure 5 shows the Kolmogorov–Smirnov tests carried out on all the numeric features (126 in total) that were common in both, Michigan and Tel-Aviv, datasets. Some extremely small $p$-values were slightly transformed, i.e., replaced by the minimum of the other non-zero $p$-values, to ensure that the logarithmic y-axis scale is correctly plotted.

False Discovery Rate (FDR) was used to control the false-positive rate at the level of 0.01. Thus, among the set of rejected null hypotheses, the expected proportion of false discoveries is limited to 1%. Assuming the tests are independent, the FDR control is achieved by calculating $q$-values (Benjamini/Hochberg FDR adjusted $p$-value^36^ for each test and rejecting those with $q$-value <0.01. The red line in Fig. 5 represents the −*log*(0.01) cutoff value.

Table 3 shows the level of similarity between Michigan and Tel-Aviv datasets in two different types of variables (clinical/demographic and neuroimaging).

**Table 3:** Some of the clinical/demographic variables and many of the neuroimaging features exhibit significantly different distributions between the two datasets.

Feature Category	Features of significant incongruence
Clinical/Demographic	24 out of 70 (34%)
Neuroimaging	54 out of 56 (96%)

**Figure 6:**
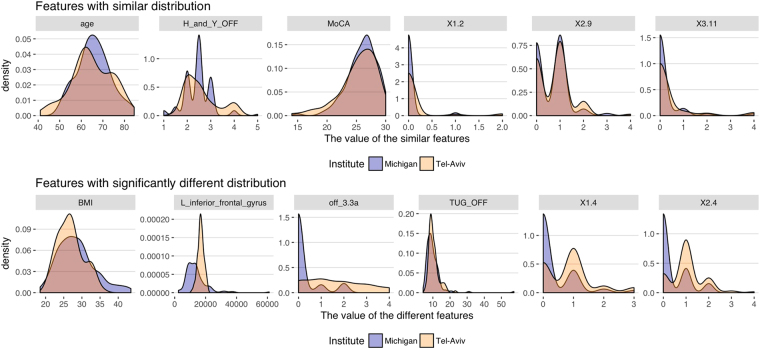
Similarities and differences between feature distributions in the Michigan and Tel-Aviv datasets.

Figure 6 includes examples of feature distributions in these two datasets showing some similarity and some differences.

As the study subjects in both Michigan and Tel-Aviv datasets represent Parkinson’s disease patients, an aggregate dataset was generated to increase the number of training and testing cases and examine the performance of the predictive analytics on the complete data. We used normalization (centering and scaling) of the data elements prior to their aggregation.

**Figure 7:**
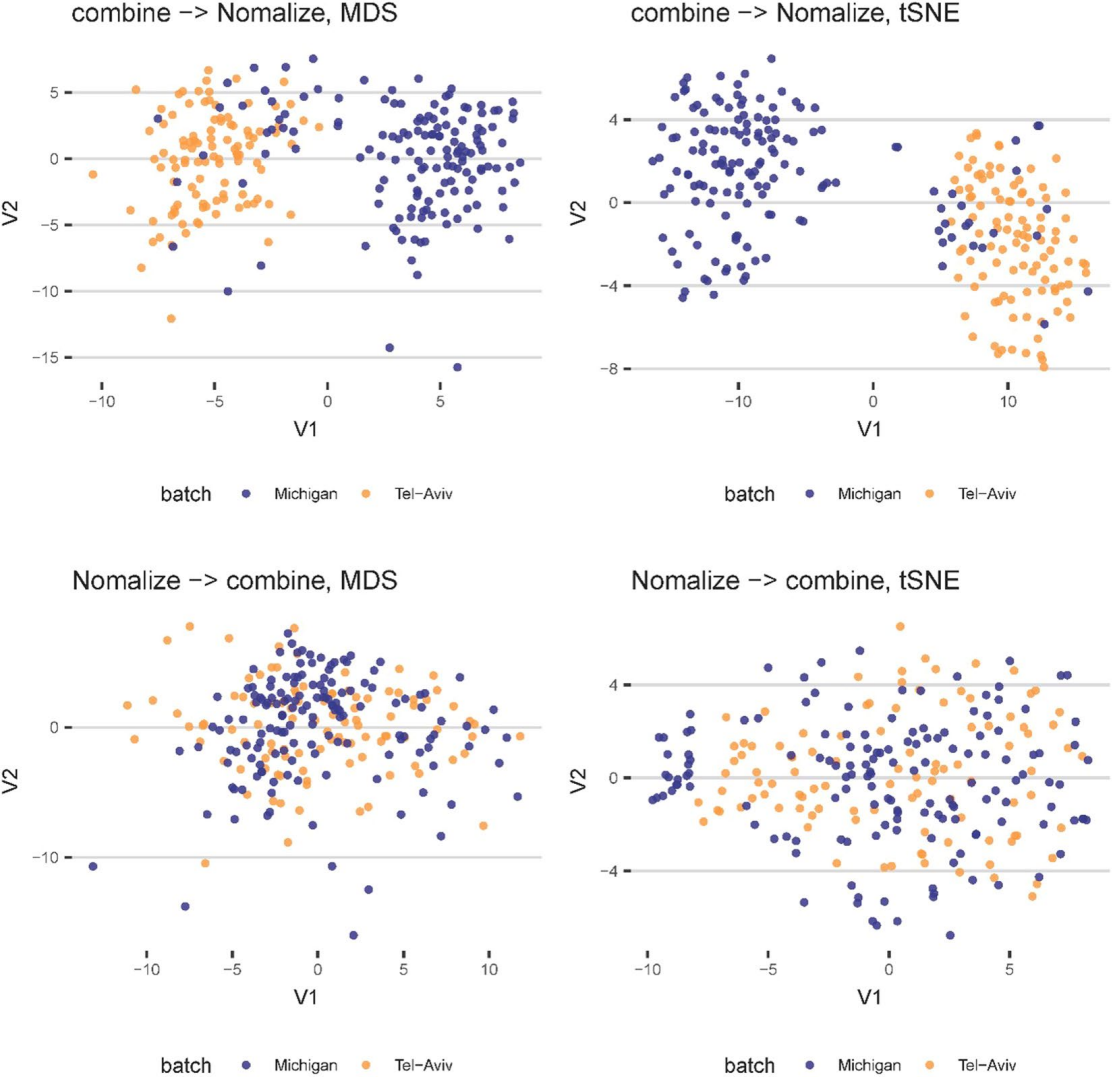
Visualization of batch effects of the aggregated data using different data aggregation strategies (normalize the two data sets separately vs. normalize the combined data) using two alternative dimensionality reduction methods - MDS (left) and tSNE (right).

Figure 7 shows batch effects on the aggregate dataset using two alternative standardization techniques – normalize two data sets separately prior to aggregation vs. aggregate and normalize the combined data. To illustrate the similarities and differences between the pair of standardization techniques we show 2D projections of the data in each paradigm (top and bottom) using both multidimensional scaling (MDS)^37^ (left) and t-distributed Stochastic Neighbor Embedding (tSNE)^32,38^ (right).

Batch effects do not represent underlying biological variability. Rather, they reflect technical sources of data variation due to handling of the samples. To untangle batch technical variation from intrinsic biomedical process variability we need to carefully select the data harmonization, normalization and aggregation strategies to avoid unintended bias. In this case, we chose to normalize each of the two datasets separately prior to their aggregation into the combined Michigan+TelAviv dataset.

#### *Challenge 2*: Identification of salient predictors associated with patients’ falls

In this part, we aim to identify for the strongest predictors for patients’ falls for each of the three datasets, Michigan, Tel-Aviv, and the aggregated Michigan+TelAviv. We carry out feature selection using two different methods: random forest (RF)^11,39^ and Knockoff filtering (KO)^40^. For each dataset, both feature selection techniques identify the top 20 selected variables. MWW test and KS test are used to compare the distributions of these features between patient subgroups (Falls vs. No-falls). We aim to identify commonly selected features by both techniques that also show significant differences on the MWW and KS tests.

### Michigan dataset

**Figure 8:**
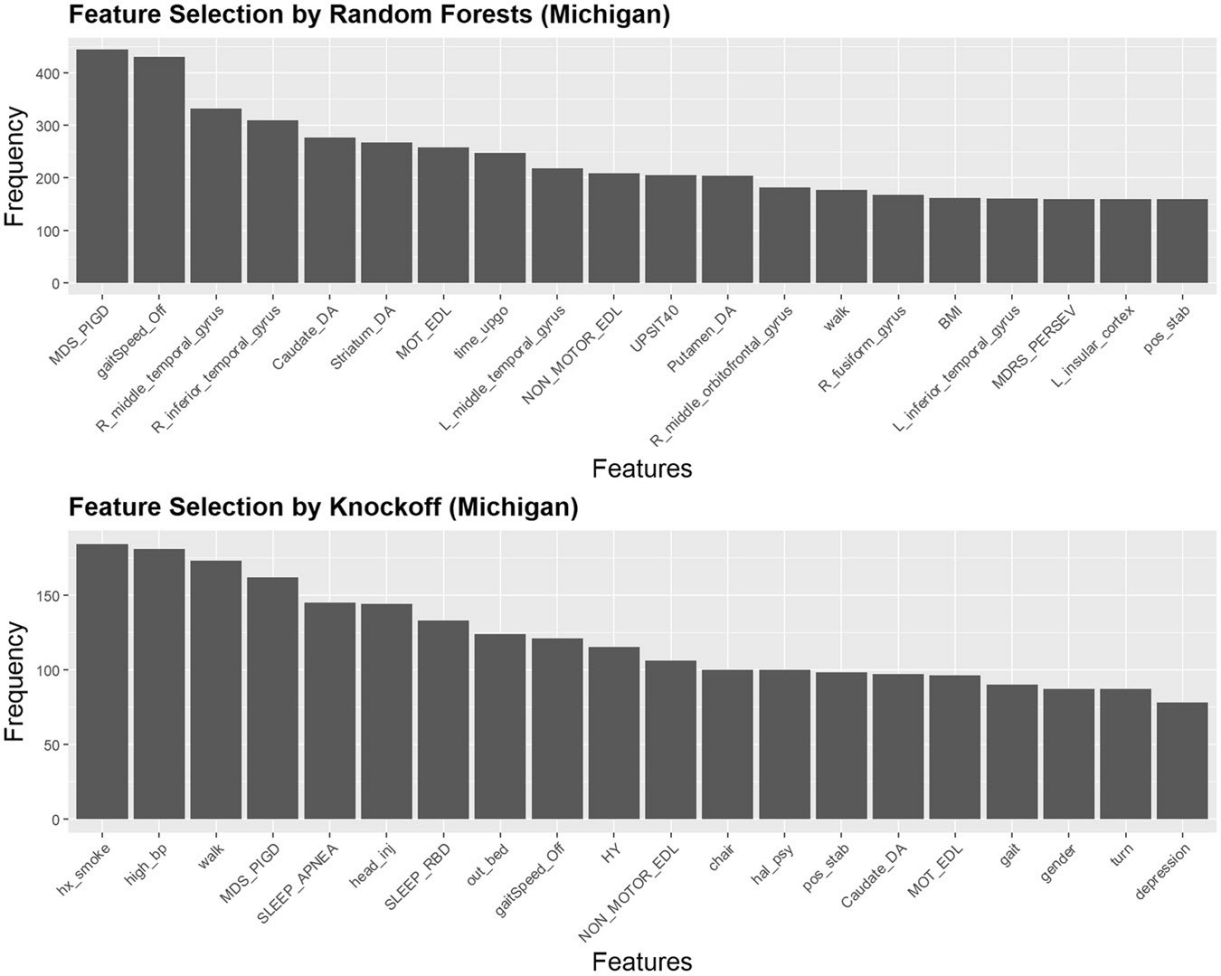
Results of feature selection for the Michigan dataset using random forest (top) and knockoff filtering (bottom). The barplots present the exact number of times the top listed features are selected.

We consider common variables selected by both LASSO^41^ and Knockoff (FDR = 0.35) as the “potentially falls-associated features”. In addition, candidate features that are significantly different on both MWW and KS tests across two cohorts (“fall” and “non-fall”) are considered “falls-associated features”. Regularized (LASSO) linear modeling rejects all genetic features, the only set of multi-level categorical features in Michigan dataset. This fact facilitates our implementation of Knockoff filtering, which is not directly applicable for multi-level categorical variables. Excluding all genetic variables, we apply Random Forest (RF) and Knockoff (KO) variable selections on all other numeric or binary features. The feature selection results are shown on Table 4 with a corresponding variable importance plots on Fig. 8. The common features selected by both methods, RF and KO, are annotated (*). The Supplementary Materials include the technical details of the two alternative feature selection strategies. RF feature section is based on fitting a number of decision trees where each node represents a single feature condition split the dataset into two branches according to an impurity measure (e.g., Gini impurity, information gain, entropy). The feature ranking reported in Table 4 reflect the frequencies that each of these top variables decreases the weighted impurity measure in multiple decision trees. KO feature selection relies on pairing each feature with a decoy variable, which resembles its characteristics but carries no signal, and optimizes an objective function that jointly estimates model coefficients and variable selection, by minimizing a the sum of the model fidelity and a regularization penalty components. The discrepancy between a real feature ($Xj$) and its decoy (knockoff) counterpart ($\tilde{X}j$) is measured by a statistic like $Wj=max(Xj,\tilde{X}j)\times sgn(Xj-\tilde{X}j)$, which effectively measures how much more important $Xj$ is relative to $\tilde{X}j$. The strength of the importance of $Xj$ relative to $\tilde{X}j$ is measured by the statistic magnitude, $|Wj|$. There is a strong evidence of the importance of the commonly selected features (*) by RF and KO, see Table 4 and Fig. 8.

**Table 4:** Feature selection for the Michigan data using RF (left) and KO (right). Six common features (*) are selected by both methods: MDS_PIGD, gaitSpeed_Off, MOT_EDL, NON_MOTOR_EDL, walk, pos_stab.

Random Forests		Knockoff	
Features	Frequency	Features	Frequency
MDS_PIGD*	0.888	hx_smoke	0.764
gaitSpeed_Off*	0.860	high_bp	0.751
R_middle_temporal_gyrus	0.662	walk*	0.718
R_inferior_temporal_gyrus	0.618	MDS_PIGD*	0.672
Caudate_DA	0.554	SLEEP_APNEA	0.602
Striatum_DA	0.534	head_inj	0.598
MOT_EDL*	0.516	SLEEP_RBD	0.552
time_upgo	0.494	out_bed	0.515
L_middle_temporal_gyrus	0.436	gaitSpeed_Off*	0.502
NON_MOTOR_EDL*	0.418	HY	0.477
UPSIT40	0.410	NON_MOTOR_EDL*	0.440
Putamen_DA	0.408	hal_psy	0.415
R_middle_orbitofrontal_gyrus	0.364	Chair	0.415
walk*	0.354	pos_stab*	0.407
R_fusiform_gyrus	0.336	Caudate_DA	0.403
BMI	0.324	MOT_EDL*	0.398
L_inferior_temporal_gyrus	0.322	gait	0.374
MDRS_PERSEV	0.320	gender	0.361
L_insular_cortex	0.318	turn	0.361
pos_stab*	0.318	depression	0.324

Table 5 shows the results comparing the distributions between fallers and no-fallers in the Michigan data, using the top six common features identified by RF and KO controlled feature selection.

**Table 5:** MWW test and KS tests of group differences performed on the commonly selected features.

Selected Features	Mann-Whitney-Wilcoxon Test		Kolmogorov-Smirnov Tests	
	W	p-value	D	p-value
MDS_PIGD	1011.5	3.933e-08	0.42934	1.935e-05
gaitSpeed_Off	3412	5.082e-06	0.37691	0.0002733
MOT_EDL	1253	8.713e-06	0.41575	3.974e-05
NON_MOTOR_EDL	1486.5	0.0005182	0.27681	0.01647
walk	1195	1.643e-07	0.41855	3.432e-05
pos_stab	1253	1.255e-06	0.37411	0.0003118

**Figure 9:**
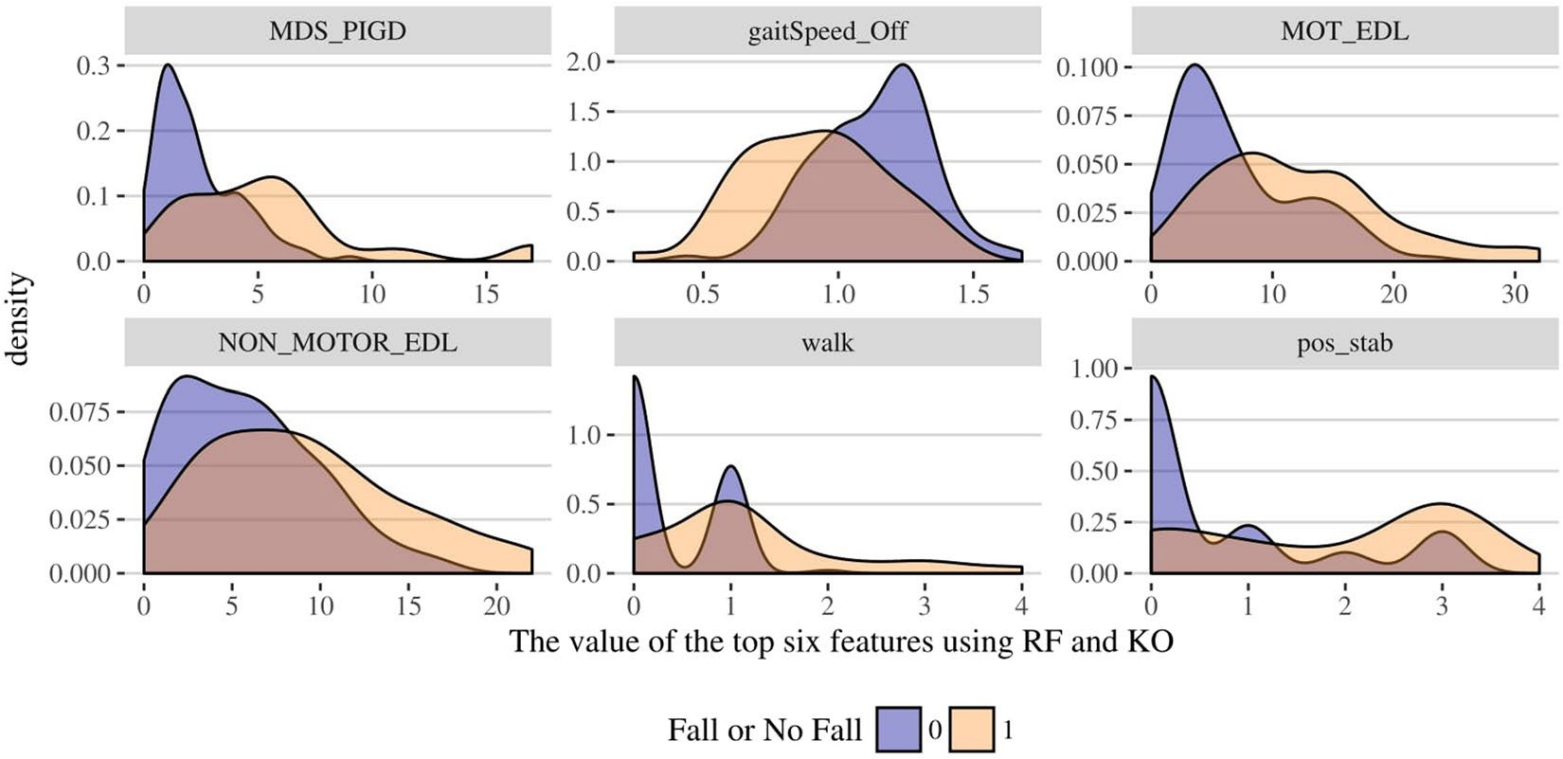
Density plots showing the top six clinical features with significantly different distributions between falls and no-fall cohorts within the Michigan study.

Figure 9 depicts the density plots of the top six selected clinical features that have significantly different distributions between falls and no-fall subpopulations in the Michigan dataset.

### Tel-Aviv data

**Figure 10:**
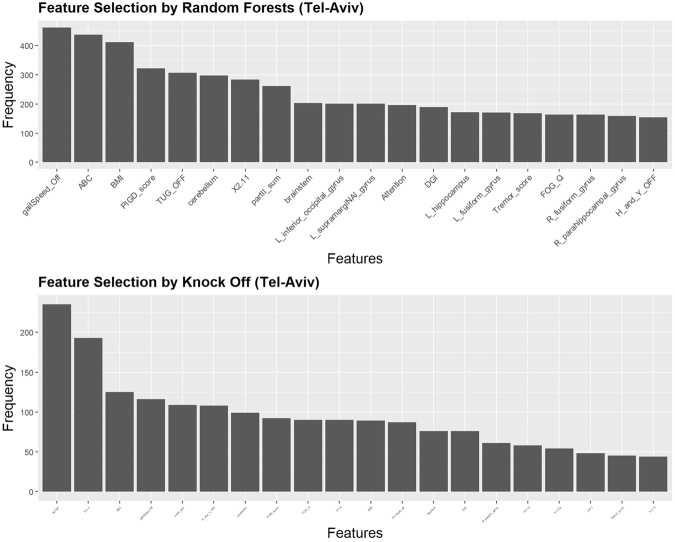
Results of feature selection for the Tel-Aviv dataset using random forest (top) and knockoff (bottom) methods. The bar plots present the exact number of times the top features are selected.

**Figure 11:**
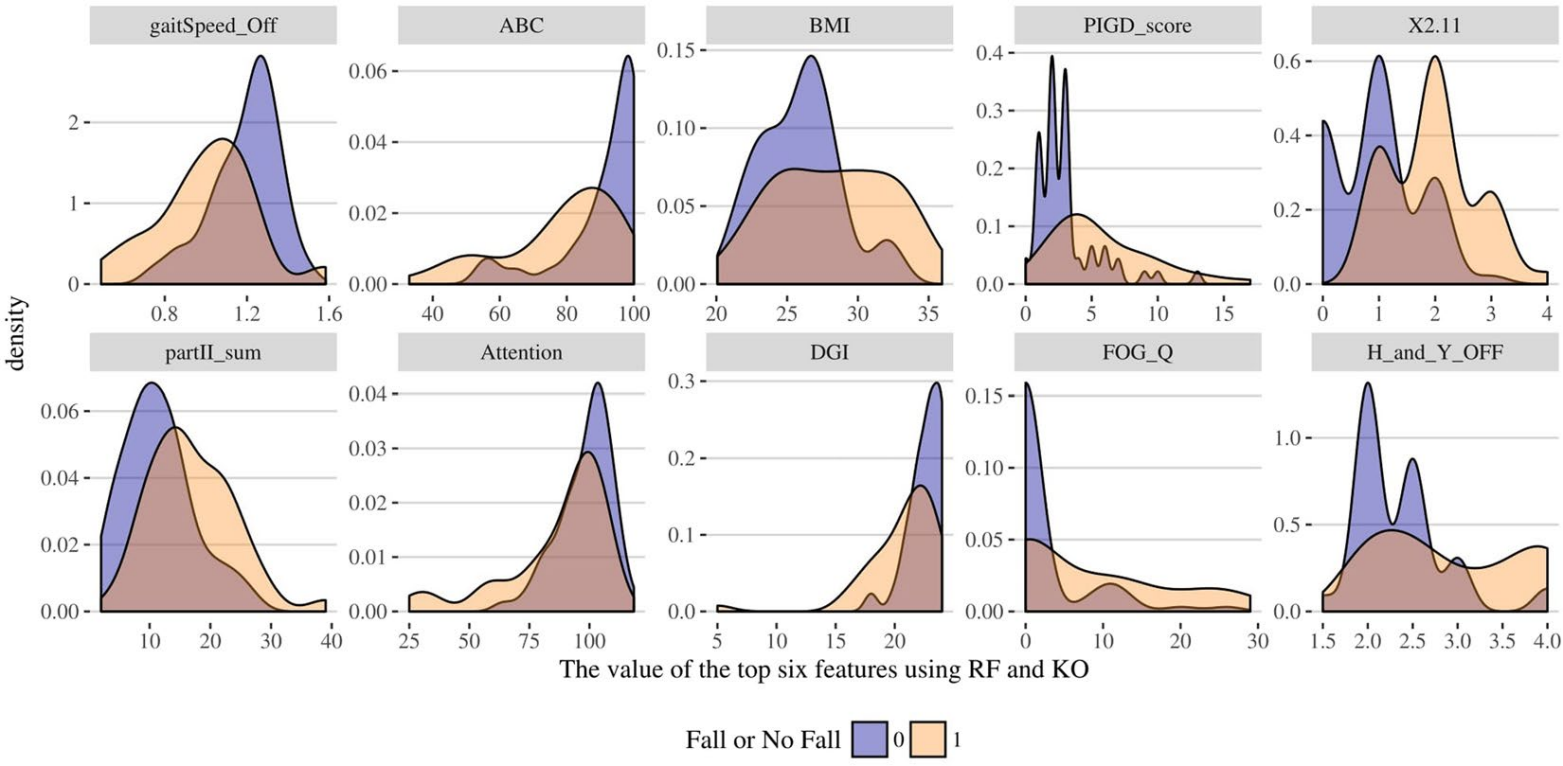
Density plots showing that the top 10 selected clinical features have significantly different distributions between falls and no-fall patient groups.

Table 6 illustrates the top features selected by RF and KO methods solely on the Tel-Aviv dataset. Again, commonly selected features by both strategies are labeled (*). Figure 10 presents the Tel-Aviv RF and KO feature selection results. Table 7 contains the MWW and KS test results comparing the distributions of fallers and no-fallers. Figure 11 shows the density plots of the top 10 selected clinical features separately for falls and no-fall groups.

**Table 6:** 13 features (*) are selected by both methods (RF and KO): gaitSpeed_Off, ABC, BMI, PIGD_score, cerebellum, X2.11, partII_sum, Attention, DGI, Tremor_score, FOG_Q, R_fusiform_gyrus, H_and_Y_OFF.

Random Forests		Knockoff	
Features	Frequency	Features	Frequency
gaitSpeed_Off*	0.924	gender	0.917
ABC*	0.874	X2.11*	0.753
BMI*	0.824	ABC*	0.488
PIGD_score*	0.644	gaitSpeed_Off*	0.452
TUG_OFF	0.614	partII_sum*	0.425
cerebellum*	0.596	H_and_Y_OFF*	0.421
X2.11	0.568	cerebellum*	0.386
partII_sum*	0.522	PIGD_score*	0.359
brainstem	0.406	FOG_Q*	0.351
L_inferior_occipital_gyrus	0.402	X1.8	0.351
L_supramargiNAl_gyrus	0.402	BMI*	0.347
Attention*	0.392	X3.10gait_off	0.339
DGI*	0.378	DGI*	0.296
L_hippocampus	0.344	Attention*	0.296
L_fusiform_gyrus	0.342	R_fusiform_gyrus*	0.238
Tremor_score*	0.336	X2.13	0.226
FOG_Q*	0.328	X3.17d	0.211
R_fusiform_gyrus*	0.328	X4.3	0.187
R_parahippocampal_gyrus	0.318	Tremor_score*	0.176
H_and_Y_OFF*	0.308	X3.13	0.172

**Table 7:** MWW test and KS test are performed on selected features in the Tel-Aviv data. Cerebellum, Tremor_score and R_fusiform_gyrus are excluded because their p-values > 0.05, for the KS test.

Selected Features	Mann-Whitney-Wilcoxon Test		Kolmogorov-Smirnov Tests	
	W	p-value	D	p-value
gaitSpeed_Off	1957	3.861e-06	0.44217	0.0001288
ABC	1977	1.927e-06	0.48308	1.988e-05
BMI	841	0.003808	0.38277	0.001447
PIGD_score	627	1.132e-05	0.47325	3.162e-05
cerebellum*	1692	0.004611	0.25374	0.06936
X2.11	490	3.008e-08	0.48151	2.143e-05
partII_sum	669.5	5.007e-05	0.37648	0.001831
Attention	1710	0.003133	0.29662	0.026
DGI	1862	4.841e-05	0.33478	0.007917
Tremor_score*	1648.5	0.01094	0.27262	0.05103
FOG_Q	802	0.0001001	0.3509	0.004586
R_fusiform_gyrus*	1665	0.008022	0.25452	0.06705
H_and_Y_OFF	752.5	0.0002507	0.34186	0.006249

### Aggregated (Michigan+TelAviv)

**Figure 12:**
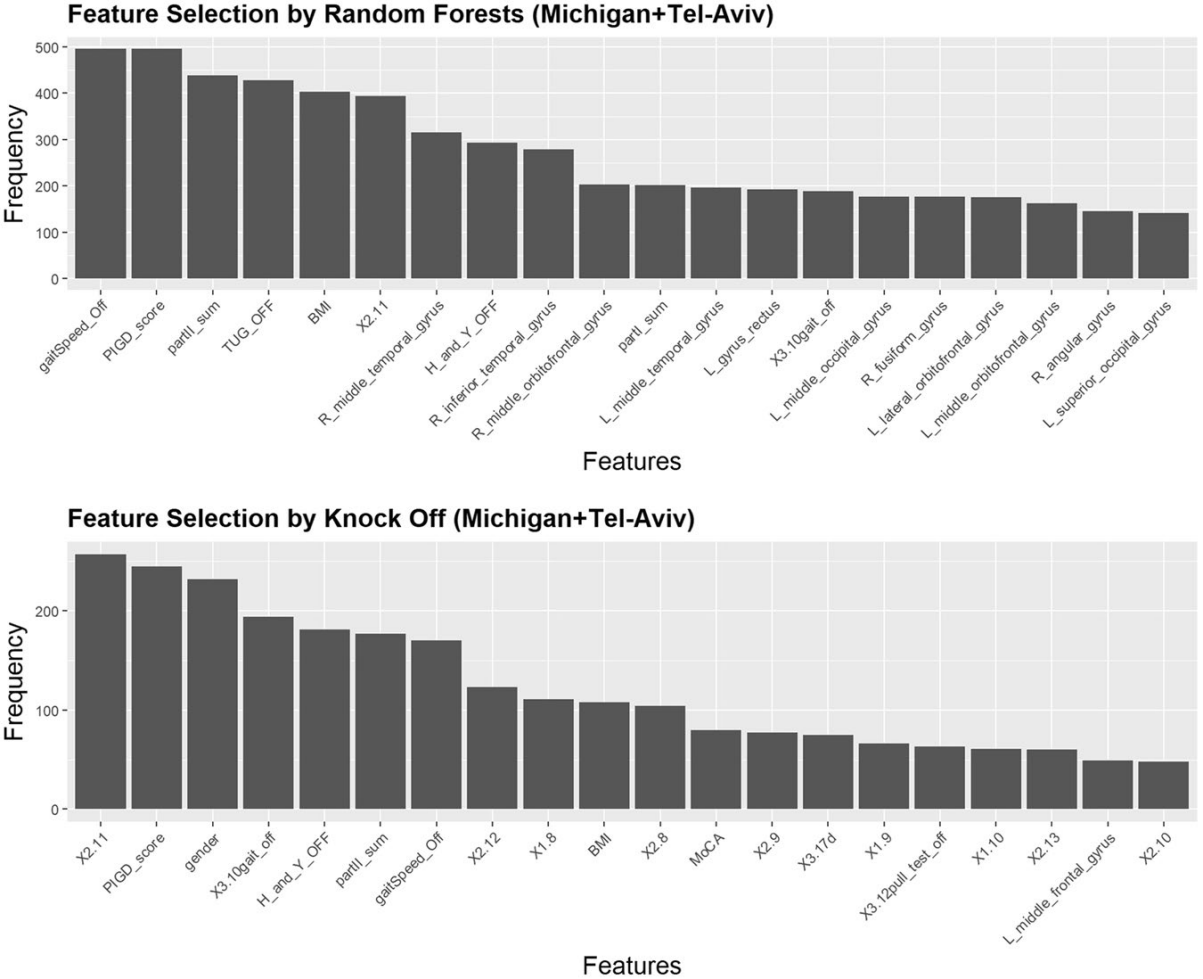
Results of feature selection for the aggregated dataset. The bar plot presents the exact number of times that the top features are selected by random forests (top) and knockoff (bottom).

**Figure 13:**
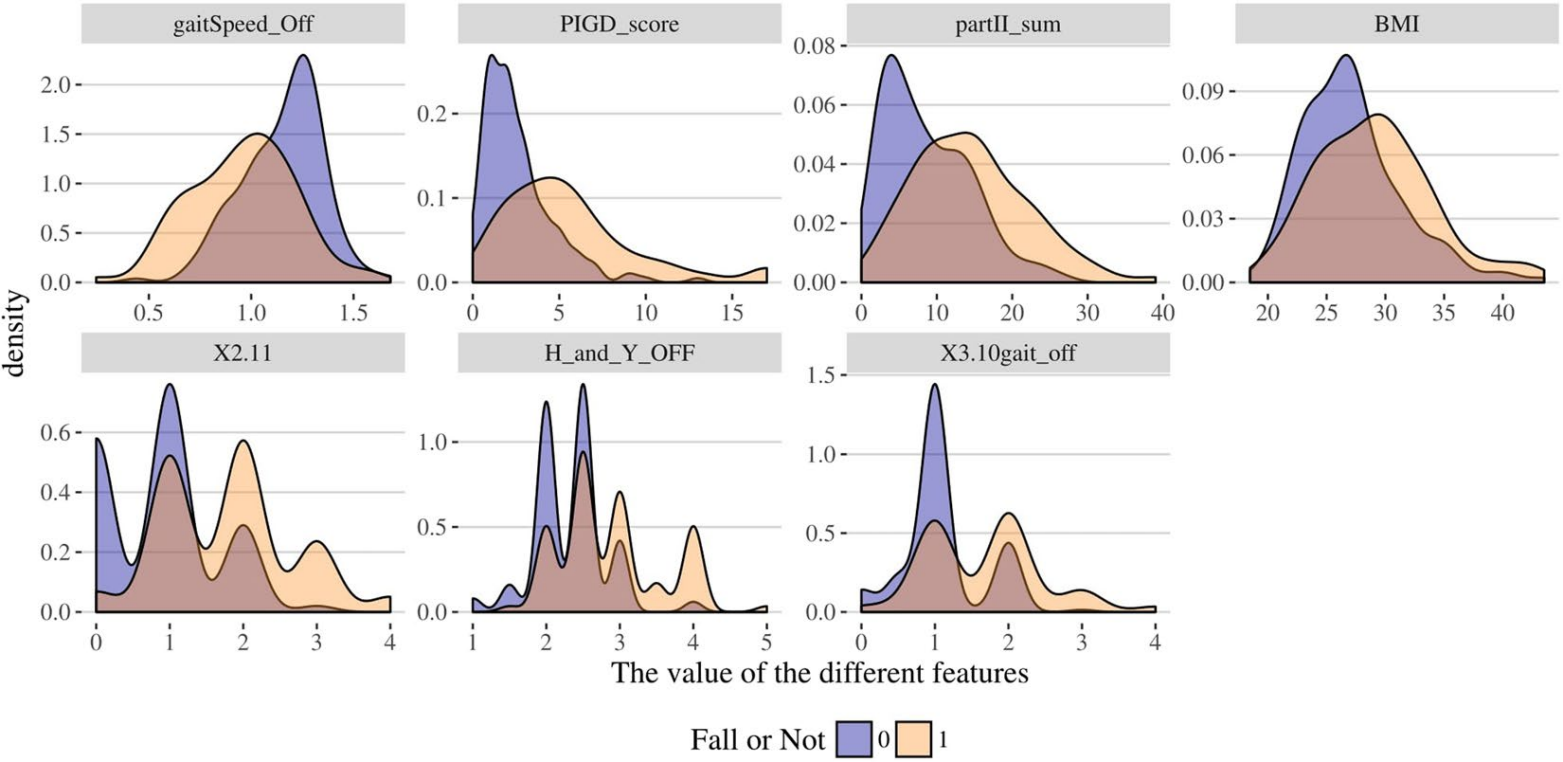
Density plots showing 7 selected clinical features with significantly different distributions between falls and no-fall groups.

Similar results, corresponding to the separate Michigan and Tel-Aviv results shown above, are included below for the aggregate Michigan+TelAviv dataset, Tables 8 and 9, Figs 12 and 13.

**Table 8:** Top seven features (*) are selected by both methods (RF and KO): gaitSpeed_Off, PIGD_score, partII_sum, BMI, X2.11, H_and_Y_OFF, X3.10gait_off.

Random Forests		Knockoff	
Features	Frequency	Features	Frequency
gaitSpeed_Off*	0.992	X2.11*	0.822
PIGD_score*	0.992	PIGD_score*	0.784
partII_sum*	0.878	Gender	0.742
TUG_OFF	0.856	X3.10gait_off*	0.621
BMI*	0.806	H_and_Y_OFF*	0.579
X2.11*	0.788	partII_sum*	0.566
R_middle_temporal_gyrus	0.632	gaitSpeed_Off*	0.544
H_and_Y_OFF*	0.586	X2.12	0.394
R_inferior_temporal_gyrus	0.558	X1.8	0.355
R_middle_orbitofrontal_gyrus	0.406	BMI*	0.346
partI_sum	0.404	X2.8	0.333
L_middle_temporal_gyrus	0.392	MoCA	0.256
L_gyrus_rectus	0.384	X2.9	0.246
X3.10gait_off*	0.376	X3.17d	0.240
L_middle_occipital_gyrus	0.354	X1.9	0.211
R_fusiform_gyrus	0.354	X3.12pull_test_off	0.202
L_lateral_orbitofrontal_gyrus	0.352	X1.10	0.195
L_middle_orbitofrontal_gyrus	0.326	X2.13	0.192
R_angular_gyrus	0.290	L_middle_frontal_gyrus	0.157
L_superior_occipital_gyrus	0.282	X2.10	0.154

**Table 9:** MWW test and KS test results for top selected features. Weight is excluded as its p-value > 0.05 in the MWW test.

Selected Features	Mann-Whitney-Wilcoxon Test		Kolmogorov-Smirnov Tests	
	W	p-value	D	p-value
gaitSpeed_Off	10442	8.745e-10	0.37139	3.373e-07
PIGD_score	3249	1.172e-12	0.44412	4.128e-10
partII_sum	3762	9.742e-10	0.36956	3.933e-07
BMI	5283.5	0.0009081	0.28083	0.0002681
X2.11	3258	9.779e-14	0.40514	1.741e-08
H_and_Y_OFF	3918	1.102e-09	0.34292	3.363e-06
X3.10gait_off	4189	3.258e-09	0.35814	1.006e-06

#### *Challenge 3*. Classification of patients’ falls

Below, we report the prediction results for the model-based logistic regression, used as a reference method, and machine learning classification using the normalized datasets. The results are reported separately for the Michigan only, Tel-Aviv only, and the aggregate Michigan+TelAviv datasets.

### Michigan data

Table 10 shows the binary classification of fall/no-fall (5-fold CV) using all features. The columns represent seven complementary performance estimating measures: accuracy (acc), sensitivity (sens), specificity (spec), positive and negative predictive values (ppv and npv), and area under the receiver operating curve (auc).

**Table 10:** Performance of model-based and model-free methods (using all features).

Method	acc	sens	spec	ppv	npv	lor	auc
Logistic Regression	0.439	0.400	0.456	0.243	0.635	−0.581	0.630
Random Forests	**0**.**764**	**0**.**356**	**0**.**942**	**0**.**727**	**0**.**770**	**2**.**188**	**0**.**727**
AdaBoost	0.703	0.333	0.864	0.517	0.748	1.156	0.695
XGBoost	0.730	0.333	0.903	0.600	0.756	1.537	0.710
SVM	**0**.**743**	**0**.**200**	**0**.**981**	**0**.**818**	**0**.**737**	**2**.**536**	**0**.**750**
Neural Network	0.655	0.444	0.748	0.435	0.755	0.863	
Super Learner	0.723	0.289	0.913	0.591	0.746	1.445	

Table 11 shows the binary classification of fall/no-fall (5-fold CV) using only the top 6 selected features (MDS_PIGD, gaitSpeed_Off, MOT_EDL, NON_MOTOR_EDL, walk, pos_stab).

**Table 11:** Performance of model-based and model-free methods (using top 6 features).

Method	acc	sens	spec	ppv	npv	lor	auc
Logistic Regression	0.736	0.289	0.932	0.650	0.750	1.718	0.781
Random Forests	**0**.**777**	**0**.**444**	**0**.**922**	**0**.**714**	**0**.**792**	**2**.**251**	0.697
AdaBoost	0.750	0.444	0.883	0.625	0.784	1.803	0.693
XGBoost	**0**.**777**	**0**.**467**	**0**.**913**	**0**.**700**	**0**.**797**	**2**.**213**	0.657
SVM	0.757	0.467	0.883	0.636	0.791	1.892	**0**.**742**
Neural Network	0.669	0.400	0.786	0.450	0.750	0.898	
Super Learner	**0**.**784**	**0**.**467**	**0**.**922**	**0**.**724**	**0**.**798**	**2**.**341**	

### Tel Aviv data

Table 12 illustrates the results of the binary classification of fall/no-fall (5-fold CV) using all features.

**Table 12:** Performance of model-based and model-free methods (using all features).

Method	acc	sens	spec	ppv	npv	lor	auc
Logistic Regression	0.505	0.390	0.581	0.381	0.590	−0.121	0.603
Random Forests	0.689	0.537	0.790	0.629	0.721	1.473	0.702
AdaBoost	**0**.**718**	**0**.**610**	**0**.**790**	**0**.**658**	**0**.**754**	**1**.**773**	**0**.**719**
XGBoost	0.670	0.610	0.710	0.581	0.733	1.340	0.711
SVM	**0**.**757**	**0**.**512**	**0**.**919**	**0**.**808**	**0**.**740**	**2**.**482**	**0**.**767**
Neural Network	0.680	0.659	0.694	0.587	0.754	1.474	
Super Learner	0.670	0.512	0.774	0.600	0.706	1.281	

Table 13 shows the binary classification of fall/no-fall (5-fold CV) using top 10 selected features (gaitSpeed_Off, ABC, BMI, PIGD_score, X2.11, partII_sum, Attention, DGI, FOG_Q, H_and_Y_OFF).

**Table 13:** Performance of model-based and model-free methods (using top 10 selected features).

Method	acc	sens	spec	ppv	npv	lor	auc
Logistic Regression	0.728	0.537	0.855	0.710	0.736	1.920	0.774
Random Forests	**0**.**796**	**0**.**683**	**0**.**871**	**0**.**778**	**0**.**806**	**2**.**677**	**0**.**821**
AdaBoost	0.689	0.610	0.742	0.610	0.742	1.502	0.793
XGBoost	0.699	0.707	0.694	0.604	0.782	1.699	0.787
SVM	0.709	0.561	0.806	0.657	0.735	1.672	**0**.**822**
Neural Network	0.699	0.610	0.758	0.625	0.746	1.588	
Super Learner	0.738	0.683	0.774	0.667	0.787	1.999	

Improving Classification Sensitivity: We attempted to further improve the classification sensitivity, which is important in this clinical setting. As Random Forest outperforms the other methods, we focused our performance tuning on RF classification. By optimizing the RF parameters, using grant weights, setting cut off points for two classes and the number of features used for each decision tree branch split, we obtained a classification model with higher sensitivity and LOR. Although, there is more room to further improvement of the sensitivity, it is also important to keep specificity within a reasonable range. Table 14 shows the best RF results on the Tel-Aviv data. Note that improving the classifier sensitivity trades off with (compromising) it’s sensitivity.

**Table 14:** Fine-tuned RF classification results on the Tel-Aviv dataset.

Method	acc	sens	spec	ppv	npv	lor
Random Forests	0.767	0.805	0.742	0.673	0.852	2.473

Fall prediction with a subset of important features: We applied a logit model for a low dimensional case-study. Our results show 74% prediction accuracy using four variables, Table 15. Prior work by Paul, *et al*.^42^ reported accuracy about 80% using three variables, including “fall in the previous year” as an additional predictor, which may be very strongly associated with the clinical outcome of interest—whether a patient is expected to fall or not.

**Table 15:** Logit model prediction of falls in the Tel-Aviv case, using only four features.

Selected Features	Acc	Sens	Spec	ppv	npv	lor
PIGD_score, FOG_Q, H&Y(OFF), gaitSpeed(Off)	**0**.**738**	**0**.**439**	**0**.**935**	**0**.**818**	**0**.**716**	**2**.**429**

**Figure 14:**
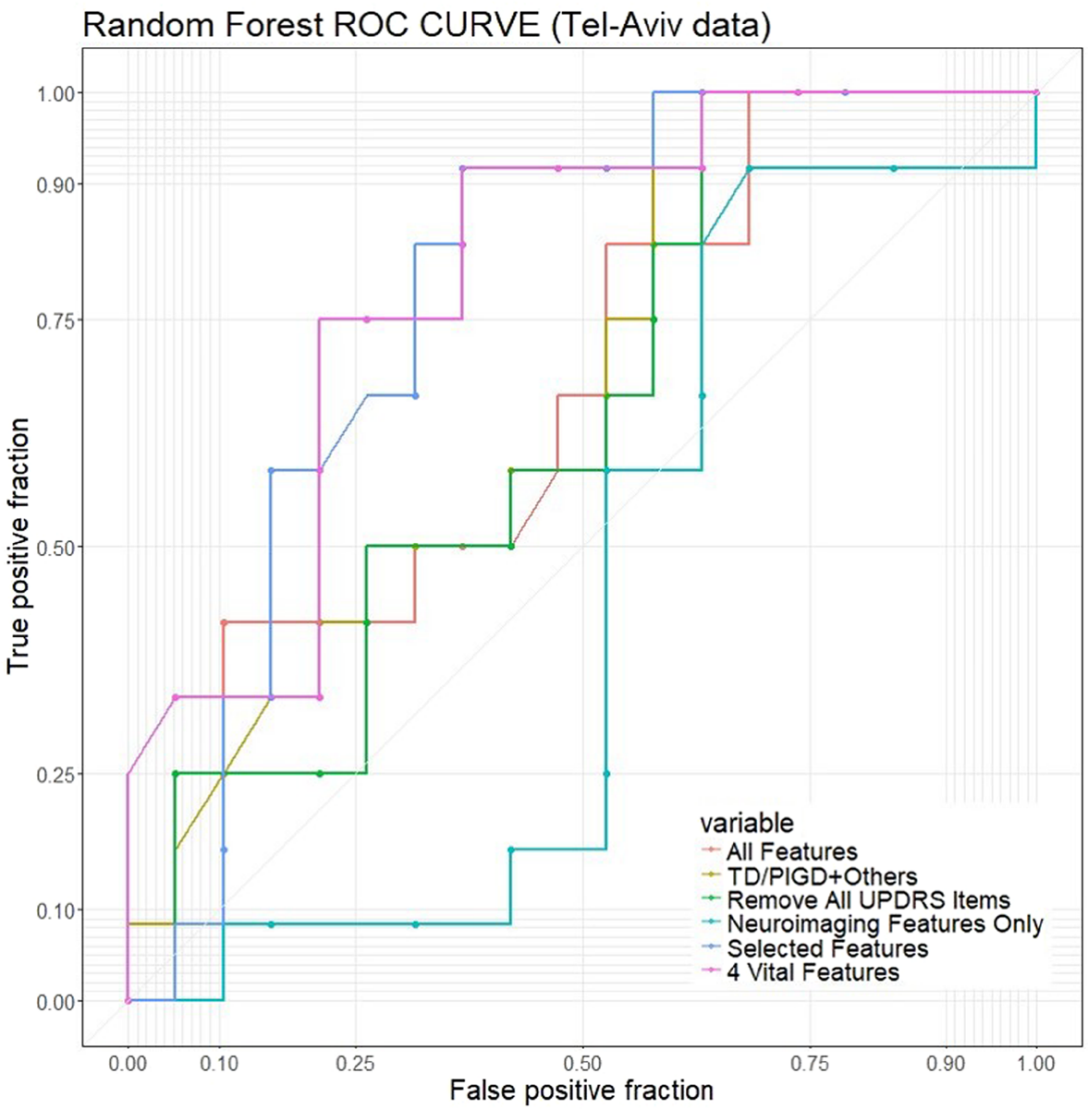
ROC plot for random Forest, lines in different colors represents the results under 6 different training conditions: (1) All features; (2) TD/PIGD classification and other clinical/demographic information; (3) Remove all UPDRS items; (4) Neuroimaging features only; (5) 10 selected features and (6) 4 vital features (PIGD Score, H_and_Y_Off, FOG_Q, gaitSpeed_Off). Corresponding Area Under the ROC Curve (AUC) are listed in Table 16.

Table 16 and Fig. 14 show the areas under the ROC curve of the Random Forest classification using several different study-designs. The results suggest that four features provide sufficient predictive power to forecast fall sin PD patients (area under the ROC curve is approximately 0.8).

**Table 16:** Performance of the RF falls/no-fall classifier under different conditions.

	All Features	TD/PIGD + Others	Remove UPDRS	Image Features	Selected Features	Four Features
AUC	0.669	0.671	0.640	0.559	0.779	0.796

Truncated classification of multiple-falls vs. no-falls (5-fold CV): A natural consideration is that some patients with prior falls might be attributed to unrelated accidents. Therefore, we tried to accurately identify patients with multiple falls. Further, for patients who had a history of falls, including one or more falls, the observations who had presence of fall by accident could mask the key demographic/clinical predictors, associated with falls. Table 17 shows the proportion of participants with two or more falls vs. no falls and Table 18 shows the classification results using all features.

**Table 17:** Distribution of patients without fall history compared to patients with two or more falls.

	no-falls	two or more falls
Number of cases (%)	62 (69%)	28 (31%)

**Table 18:** Performance of model-based and model-free methods (using all features) for Tel-Aviv dataset to predict no fall or at least two falls, contrast to results in Table 10 (fall/no-fall), using the same features.

Method	acc	sens	spec	ppv	npv	lor	auc
Random Forests	**0**.**767**	**0**.**464**	**0**.**903**	**0**.**684**	**0**.**789**	**2**.**090**	0.821
AdaBoost	**0**.**789**	**0**.**536**	**0**.**903**	**0**.**714**	**0**.**812**	**2**.**377**	0.836
XGBoost	0.711	0.393	0.855	0.550	0.757	1.338	**0**.**848**
SVM	**0**.**733**	**0**.**643**	**0**.**774**	**0**.**563**	**0**.**828**	**1**.**820**	**0**.**839**
Neural Network	**0**.**733**	**0**.**679**	**0**.**758**	**0**.**559**	**0**.**839**	**1**.**889**	
Super Learner	0.744	0.393	0.903	0.647	0.767	1.798	

Finally, Table 19 shows the classification using only the commonly selected features.

**Table 19:** Performance of model-based and model-free methods (using selected features) for Tel-Aviv dataset to predict no fall or at least two falls, contrast to results in Table 11 (falls/no-fall).

Method	acc	sens	spec	ppv	npv	lor	auc
Random Forests	**0**.**811**	**0**.**714**	**0**.**855**	**0**.**690**	**0**.**869**	**2**.**689**	**0**.**880**
AdaBoost	**0**.**822**	**0**.**750**	**0**.**855**	**0**.**700**	**0**.**883**	**2**.**872**	**0**.**886**
XGBoost	0.811	0.643	0.887	0.720	0.846	2.649	**0**.**885**
SVM	**0**.**833**	**0**.**714**	**0**.**887**	**0**.**741**	**0**.**873**	**2**.**978**	**0**.**881**
Neural Network	0.722	0.607	0.774	0.548	0.814	1.667	
Super Learner	0.800	0.643	0.871	0.692	0.844	2.497	

The best results were obtained using adaptive boosting (Adaboost)^12^ and SVM with Gaussian kernel^43^.

### Aggregate Michigan + TelAviv Data

Table 20 shows the binary falls/non-fall classification of the mixed/aggregated data using all features (5-fold CV).

**Table 20:** Performance of model-based and model-free methods (using all features) on aggregated data.

Method	acc	sens	spec	ppv	npv	lor	auc
Logistic Regression	0.594	0.488	0.648	0.420	0.709	0.566	0.639
Random Forests	**0**.**737**	**0**.**407**	**0**.**909**	**0**.**700**	**0**.**746**	**1**.**926**	**0**.**772**
AdaBoost	0.717	0.407	0.879	0.636	0.740	1.605	0.753
XGBoost	0.689	0.419	0.830	0.563	0.733	1.259	0.734
SVM	0.629	0.558	0.667	0.466	0.743	0.927	**0**.**768**
Neural Network	0.641	0.488	0.721	0.477	0.730	0.904	
Super Learner	**0**.**729**	**0**.**430**	**0**.**885**	**0**.**661**	**0**.**749**	**1**.**758**	

Table 21 illustrates the results of the mixed/aggregated data (5-fold CV) classification using only the seven commonly selected features: gaitSpeed_Off, PIGD_score, partII_sum, BMI, X2.11, H_and_Y_OFF, X3.10gait_off.

**Table 21:** Performance of model-based and model-free methods (using selected features) on aggregated data.

Method	acc	sens	spec	ppv	npv	lor	auc
Logistic Regression	**0**.**773**	**0**.**430**	**0**.**952**	**0**.**822**	**0**.**762**	**2**.**696**	**0**.**817**
Random Forests	0.705	0.453	0.836	0.591	0.746	1.445	0.774
AdaBoost	**0**.**717**	**0**.**558**	**0**.**800**	**0**.**593**	**0**.**776**	1.620	0.765
XGBoost	**0**.**745**	**0**.**547**	**0**.**848**	**0**.**653**	**0**.**782**	1.909	0.781
SVM	0.777	0.512	0.915	0.759	0.782	2.425	**0**.**785**
Neural Network	0.661	0.512	0.739	0.506	0.744	1.089	
Super Learner	0.729	0.453	0.873	0.650	0.754	1.739	

Train on Michigan and Test on Tel-Aviv Data: Table 22 shows the falls/no-fall classification (training on Michigan and testing on Tel-Aviv data) results using the selected features.

**Table 22:** Performance of model-based and model-free methods. Train on Michigan and test on Tel-Aviv data.

Method	acc	sens	spec	ppv	npv	lor	auc
Logistic Regression	0.718	0.390	0.935	0.800	0.699	2.228	**0**.**832**
Random Forests	**0**.**738**	**0**.**537**	**0**.**871**	**0**.**733**	**0**.**740**	**2**.**056**	0.796
AdaBoost	0.699	0.463	0.855	0.679	0.707	1.626	0.791
XGBoost	0.709	0.463	0.871	0.704	0.711	1.763	0.758
SVM	0.689	0.268	0.968	0.846	0.667	2.398	**0**.**827**
Neural Network	0.631	0.585	0.661	0.533	0.707	1.014	
Super Learner	**0**.**757**	**0**.**562**	**0**.**887**	**0**.**767**	**0**.**753**	**2**.**31**	

#### Train on Tel-Aviv and Test on Michigan Data

Table 23 shows the opposite falls/no-fall classification (training on Tel-Aviv and testing on Michigan data) results using only the commonly selected features.

**Table 23:** Performance of model-based and model-free methods. Train on Tel Aviv and test on Michigan.

Method	acc	sens	spec	ppv	npv	lor	auc
Logistic Regression	**0**.**777**	**0**.**489**	**0**.**903**	**0**.**688**	**0**.**802**	**2**.**186**	**0**.**794**
Random Forests	**0**.**709**	**0**.**667**	**0**.**728**	**0**.**517**	**0**.**833**	**1**.**678**	0.755
AdaBoost	0.689	0.644	0.709	0.492	0.820	1.484	0.780
XGBoost	0.730	0.600	0.786	0.551	0.818	1.709	0.748
SVM	**0**.**797**	**0**.**444**	**0**.**951**	**0**.**800**	**0**.**797**	**2**.**752**	**0**.**805**
Neural Network	0.622	0.644	0.612	0.420	0.797	1.049	
Super Learner	**0**.**770**	**0**.**644**	**0**.**825**	**0**.**617**	**0**.**842**	**2**.**15**	

### *Challenge 4*. Morbidity phenotype (TD/PIGD) Classification

Next, ignoring the UPDRS subitems, we performed predictive analytics of tremor dominant (TD) vs. posture instability and gait disorder (PIGD) classification using only the demographic and clinical information (neuroimaging features were excluded).

### Michigan Data

Table 24 shows that compared to prediction of falls using all features, the overall accuracy for both logistic regression and AdaBoost TD/PIGD classification is improved, compare to Table 10.

**Table 24:** Performance of prediction for TD/PIGD class label on Michigan dataset.

Method	acc	sens	spec	ppv	npv	lor
Logistic Regression	0.615	0.311	0.748	0.350	0.713	0.291
Random Forests	**0**.**743**	**0**.**356**	**0**.**913**	**0**.**640**	**0**.**764**	**1**.**751**
AdaBoost	0.743	0.422	0.883	0.613	0.778	1.712

### Tel-Aviv Data

Table 25 demonstrated improved sensitivity of TD/PIGD classification, as compared to prediction of falls using all features (Table 12). This indicates TD/PIGD classification may also be an important predictor of patients’ falls.

**Table 25:** Performance of prediction for TD/PIGD class label on Tel-Aviv dataset.

Method	acc	sens	spec	ppv	npv	lor
Logistic Regression	0.738	0.707	0.758	0.659	0.797	2.024
Random Forests	**0**.**738**	**0**.**610**	**0**.**823**	**0**.**694**	**0**.**761**	**1**.**980**
AdaBoost	0.728	0.634	0.790	0.667	0.766	1.877

### Aggregated Michigan+TelAviv Data

Table 26 shows a slightly higher sensitivity for random forest and AdaBoost TD/PIGD classification, compared to falls prediction using all features (Table 20). Yet, compared to within archive training with internal CV assessment, the performance of both classifiers on the aggregated dataset is less impressive, which may be explained by the heterogeneity of the sets discussed in Challenge 1.

**Table 26:** Performance of the prediction of TD/PIGD label on the aggregated dataset.

Method	acc	sens	spec	ppv	npv	lor
Logistic Regression	0.713	0.279	0.939	0.706	0.714	1.792
Random Forests	0.689	0.430	0.824	0.561	0.735	1.264
AdaBoost	0.713	0.477	0.836	0.603	0.754	1.538

## Discussion and Conclusions

Regarding *Challenge 1* (*data compilation*), we carefully examined, harmonized and aggregated the two independently acquired PD datasets. The merged dataset was used to retrain the algorithms and validate their classification accuracy using internal statistical cross validation. The substantial biomedical variability in the data may explain the fact that the predictive accuracy of the falls/no-fall classification results were lower in the merged aggregated data compared to training and testing the forecasting methods on each dataset separately.

*Challenge 2* (*feature selection for prediction of falls*) showed that three variables appear to be consistently chosen in the feature selection process across Michigan, Tel-Aviv and aggregated datasets – the MDS-UPDRS PIGD subscore (MDS_PIGD), gait speed in the off state, and sum score for MDS-Part II: Motor Aspects of Experiences of Daily Living (M-EDL). This is consistent with expectations as PIGD has been previously related to fall risk in PD.

In the *third Challenge* (*prediction of falls*), we found some differences between the classification results obtained by training of the three different datasets. For instance, training on the Michigan data, the highest overall classification accuracy was about 78%, with a lower sensitivity, ~47%. Whereas, training on the Tel-Aviv data, the accuracy and sensitivity rates reached 80% and 68%, respectively. For the Tel-Aviv data, the prediction model can be tuned to yield a sensitivity of 81% and accuracy of 77%. Furthermore, training on the Tel-Aviv data yields better results when the classification outcome corresponds to discriminating PD patients with multiple falls from those without falls. When training on the aggregated dataset, the falls/no-fall classification accuracy is about 70% with sensitivity around 55%. The most realistic, yet difficult, case involves external out-of-bag validation, training on one of the datasets and testing on the other. For instance, training an RF classifier on the Tel-Aviv dataset and tested it out of-bag on the Michigan dataset yields accuracy of 71% and sensitivity of 67%.

The results of the *last Challenge* (*TD/PIGD*) suggest that tremor dominant (TD) vs. postural instability and gait difficulty (PIGD) classification is reliable. For example, training and statistically validating on the Tel-Aviv data yields accuracy of 74%, sensitivity of 61% and specificity of 82%.

The classification performance of different machine learning methods varies with respect to the testing and training datasets. Overall, the random forests classifier works best on most combinations of training/testing datasets and feature selection strategies. The boosting method also showed high predictive classification accuracy on Tel-Aviv data. When the number of features is small, logistic regression may provide a viable model for predicting patient falls and it has always the benefit of easy intuitive interpretation within the scope of the problem.

The reported variable importance results may be useful for selecting features that may be important biomarkers helping clinicians quantify the risk of falls in PD patients. This study may have some potential pitfalls and limitations. For instance, the sample sizes are relatively small, Michigan (N_1_ = 148) and Tel-Aviv (N_2_ = 103). There was significant heterogeneity of the feature distributions between the Michigan and Tel-Aviv datasets. It is not clear if there were underlying biological, clinical, physiological, or technological reasons for the observed variation. This is a common challenge in all Big data analytic studies relying on multisource heterogeneous data. Features that were completely incongruent between the two data archives were removed from the subsequent analyses and were not included in the aggregated dataset. Finally, the classifiers trained on one of the datasets (Tel-Aviv) performed better when tested either via internal statistical cross-validation or via external out-of-bag valuation (using the Michigan test data). Our study of falls primarily focused on the binary indicator of falls. The frequency of falls, or the severity of falls, were not examined due to lack of sufficient information in either data archive. However, both frequency and severity of falls require further examination.

### Clinical impact

The study findings indicate that clinical markers of PIGD motor features were more robust predictors of falls than striatal dopamine bindings as measured by DTBZ VMAT2 brain PET imaging. Along the same line, typical clinical predictors of nigrostriatal dopaminergic losses, such as distal bradykinesias did not significantly predict falls in the analyses. These findings underscore the notion that falls are more related to extra-striatal and non-dopaminergic mechanisms than striatal dopamine level per se. The presented results suggest a need for new approaches for determining fall risk and motor phenotypes among patients with PD. If the conclusions are replicated on a larger scale and reproduced in prospective studies, then the methods described here can contribute to the diagnosis and prognosis, and perhaps to personalized or individualized treatment approaches.

### Synergies with previous studies

We have previously shown that PD fallers did not differ in nigrostriatal dopaminergic nerve terminal integrity but had lower cholinergic brain activity compared to the PD no-fallers^44,45^. We have also shown in prior analyses that freezing of gait is most prominent with extra-striatal non-dopaminergic changes, in particular the combined presence of cholinergic denervation and β-amyloid plaque deposition^46^. Some of the clinical predictors of falls in this study, such as slow gait speed or PIGD motor feature severity have been found to associate with cortical cholinergic and β-amyloid plaque deposition, respectively^47,48^ and were independent from the degree of nigrostriatal nerve terminal losses.

Another interesting observation in our analyses is that brain MRI morphometry measures did not appear to be robust predictors of fall status. It should be noted that mobility functions are subserved by a widespread network of interconnected brain and extra-cranial structures (e.g., spinal cord, nerves). Therefore, it is unlikely that individual brain structures may be highly salient predictive features. In this study, infratentorial brain structures, such as the cerebellum and brainstem, performed relatively better than supratentorial brain regions. Another factor is that the etiology of falls is multi-factorial (cognitive impairment, freezing of gait, sarcopenia, postural instability) and thereby involving multiple neural and neuromuscular structures and connections. It is plausible, however, that more precise clinical sub-typing of specific fall mechanisms, may identify more vulnerable brain regions or networks of regions.

There are enormous opportunities for expanding this work to include additional classifiers, explore alternative features, validate on new cohorts and translate into clinical practice. For example, utilizing novel computational models and genomic biomarkers (e.g., noncoding RNA) may improve the automated PD diagnosis. For example, publicly available archives including long noncoding RNAs^49,50^, micro RNAs^51,52^, or other sequence, expression, or functional data may provide additional power to reduce classification error and enhance the forecasting reproducibility. Extreme Gradient Boosting Machine or other powerful classifiers may be able to improve the diagnostic prediction by capitalizing on RNA functional similarity, disease semantic similarity, and other RNA-disease associations^53^. Knowledge-based machine learning is an alternative strategy for disease classification^54^. Combinatorial genomic signature sets^55^ and molecular signaling networks^56,57^ may also be useful to predict, prognosticate, or forecast motor and cognitive decline in PD. In addition, combining these approaches with metrics extracted from long-term (e.g., 24/7) monitoring of movement also holds promise for enhancing this line of work^21,58^.

The present transdisciplinary work illustrates some of the advantages of open-science principles, collaborative research, and independent validation of findings. We have compiled and are sharing the entire data preprocessing pipeline, visualization tools, and analytic protocol. This promotes community-wide validation, improvements, and collaborative transdisciplinary research into other complex healthcare and biomedical challenges. The R-based Predictive Analytics source-code is released under permissive LGPL license on our GitHub repository (https://github.com/SOCR).

## Electronic supplementary material

Supplementary Materials (Appendix)
