# Supplementary material for: Model-based and Model-free Machine Learning Techniques for Diagnostic Prediction and Classification of Clinical Outcomes in Parkinson’s Disease
Source: Sci Rep. 2018 May 8;8:7129. doi: 10.1038/s41598-018-24783-4 (PMC5940671; doi:10.1038/s41598-018-24783-4)
Supplement: Supplementary file 1 — Supplementary Materials (Appendix) [file 41598_2018_24783_MOESM1_ESM.docx]

**Supplementary Materials**

Model-based and Model-free Machine Learning Techniques for Diagnostic Prediction and Classification of Clinical Outcomes in Parkinson’s Disease

Chao Gao ^1,2^, Hanbo Sun ^1,3^, Tuo Wang ^1,3^, Ming Tang ^1,2^ , Nicolaas I. Bohnen ^4,5,6^,

Martijn L.T.M. Müller ^4,5,6^, Talia Herman ^7^, Nir Giladi ^7,9^, Alexandr Kalinin ^1,6,11^,

Cathie Spino ^2,6^, William Dauer ^5,6^, Jeffrey M. Hausdorff ^7,8,10^, Ivo D. Dinov ^1,6,11,12 *^

### I. Methods

### **I.1 Datasets**

**I.1.a Michigan Data, additional details**

*Inclusion criteria:*

1. Age 50 and above (M/F).
2. PD diagnosis based on the UK Parkinson's Disease Society Brain Bank Research Center (UKPDSBRC) clinical diagnostic criteria for PD [1]. Modified Hoehn and Yahr stages 1-4 [2]. Subjects can take dopaminergic drugs, Catechol-O-Methyl-Transferase (COMT) inhibitors, or monoamine oxidase-B (MAO-B) inhibitors. Absence of dementia and MCI confirmed by neuropsychological testing.
3. All PD subjects were required to have nigrostriatal denervation as demonstrated by DTBZ PET imaging.

*Exclusion Criteria:*

1. Subjects meeting criteria for dementia with Lewy bodies (DLB).
2. Other disorders which may resemble PD with or without demen­tia, such as vascu­lar dementia, normal pressure hydrocephalus, progressi­ve supranuclear palsy, multiple system atrophy, corticobasal ganglionic dege­neration, or toxic causes of parkinsonism. Prototypical cases have distincti­ve clinical profiles, like vertical supranuclear gaze palsy, early and severe dysautonomia or appendicular apraxia, which may differentiate them from idiopathic PD. The use of the UKPDSBRC clinical diagnostic criteria for PD and requirement of nigrostriatal denervation on DTBZ PET will mitigate the inclusion of patients with atypical parkinsonism.
3. Subjects on neuroleptic, tricyclic antidepressants, trazodone, modafinil, psychostimulants, anticholinergic (trihexiphenidyl, benztropine), or cholinesterase inhibitor drugs.
4. Evidence of a stroke in a clinically relevant area (cerebral cortex, basal ganglia, thalamus) or mass lesion on structural brain imaging (MRI or CT).
5. Participants in whom MRI is contraindicated including, but not limited to, those with a pacemaker, presence of metallic fragments near the eyes or spinal cord, or cochlear implant.
6. Severe claustrophobia precluding MR (or PET) imaging
7. Subjects limited by previous participation in research procedures involving ionizing radiation.
8. Pregnancy (test within 48 hours of each PET session) or breastfeeding
9. History of mental retardation.
10. History of deep brain stimulation (DBS) surgery.

The raw dataset compiled at Michigan contains study subjects’ demographics, PET, behavior_sensory, Mattis Dementia, sleeping, genetics, number of falls, clinical measures and neuroimaging (207 variables in total). Among the 225 study subjects, there were 148 patients with Parkinson’s disease and 77 healthy participants. Among those asymptomatic controls, only 3 had a history of falls, which provided little information about the association between falls and other covariates in the controls. Initially, we tried to rebalance the cohorts by using the synthetic minority over-sampling technique [3]. However, the results turned out to be unreliable, due to the extremely limited sample-size of the control fallers. Hence, we excluded the controls from the falls analysis. During the data cleaning process, several variables were removed due to duplication, extreme missingness, and unbalanced class labels. Multiple imputation using chained equations was to obtain complete data (m=5, 5 imputed datasets) [4]. Numeric and categorical missing values were replaced by group medians and random sampling of the imputed values. Binary variable for falls was derived (“0” for patients without fall history vs. “1” for patients with one or more falls). Patients are divided into two subtypes: tremor dominant (TD) and postural instability gait difficulty (PIGD) based on *Stebbins et al.,*  [5]. MDS_TREM and MDS_PIGD are corresponding sum scores, **Table S.1**. Gaitspeed is obtained using the following formula: gait_speed (Off) = 8.5/time_walk. To remove measuring units, all numerical variables were centered around the mean and scaled by the standard deviation. In the end, the processed Michigan dataset consisted of 170 variables for 148 PD patients.

**TableS.1**: Michigan data dictionary.

| age | age | | | |
| --- | --- | --- | --- | --- |
| gender | gender | | | |
| duration | duration of PD motor disease | | | |
| LED | levodopa equivalent dose | | | |
| mds_body | clinically most affected body side | | | |
| MOCA | Montreal cognitive assessment | | | |
| HY | Hoehn and Yahr stage of PD | | | |
| MDS_MOT | Movement Disorders Society Revised Unified Parkinson's disease rating scale total motor examination score | | | |
| NON_MOTOR_EDL | Movement Disorders Society Revised Unified Parkinson's disease rating scale non-motor experiences of living score | | | |
| MOT_EDL | Movement Disorders Society Revised Unified Parkinson's disease rating scale motor experiences of living score | | | |
| MDS_TREM | Movement Disorders Society Revised Unified Parkinson's disease rating scale tremor score | | | |
| MDS_PIGD | Movement Disorders Society Revised Unified Parkinson's disease rating scale postural instability gait difficulties score | | | |
| MDS_RIGID | Movement Disorders Society Revised Unified Parkinson's disease rating scale rigidity score | | | |
| MDS_BRADY | Movement Disorders Society Revised Unified Parkinson's disease rating scale bradykinesia score | | | |
| MDS_motor_phenotype | Movement Disorders Society Revised Unified Parkinson's disease rating scale motor phenotype (tremor-predominant, PIGD, indeterminate) | | | |
| time_upgo | timed up and go test | | | |
| time_walk | time walking 8.5 meters | | | |
| Striatum_DA | | C11 DTBZ | total striatal dopamine binding |  |
| Putamen_DA | | C11 DTBZ | putamen dopamine binding |  |
| Caudate_DA | | C11 DTBZ | caudate dopamine binding |  |
| UPSIT | | University of Pennsylvania Smell Identification Test (40 odors) | |  |

**I.1.b Tel-Aviv Data, additional details**

Additional studies analyzed gray matter changes, changes in connectivity as studied using MRI, and the relationship with freezing of gait, falls and motor phenotypes [6]. In addition to walking in the lab, subjects wore a body-fixed sensor for three days to examine daily behavior. Study of this data revealed differences between freezers and non-freezers and associations with fall risk [7-9]. Finally, a recent follow-up study in a subset of subjects examined changes over a 5 year period in motor and cognitive function [10]. Participants were studied on two separate occasions. The first visit included a neurological and clinical examination. Several tests were first performed in the "off" medication state (i.e., at least 12 hours after intake of anti-parkinsonian medications). Subsequently, patients took their usual morning medications and the evaluation continued when the patients reached their "on" state. The MRI scan was performed on a separate visit that took place within 2 weeks of the clinical exam.

*Study Participants*: 110 patients with idiopathic PD were recruited from our databases, referrals from specialists at the outpatient movement disorders unit, and from other affiliated clinics. Subjects were included if they were diagnosed by a movement disorders specialist as having idiopathic PD (as defined by the UK Brain Bank criteria), were between 40 and 85 of age, and were not demented. Subjects were excluded if they had brain surgery in the past including implanted deep brain stimulation or had significant co-morbidities likely to affect gait, e.g., acute illness, orthopedic disease, or history of stroke. In addition, subjects who could not walk independently in the off medication cycle, patients with claustrophobia, and patients who could not undergo MRI testing (e.g., if they had large metal implants) were excluded.

*Classification into PIGD and TD motor subtypes*: Initially, patients were classified into PIGD, TD or indeterminate groups using the approach proposed in 1990 by Jankovic et al., [11]. Based on the original Unified Parkinson's Disease Rating Scale (UPDRS), we computed a mean tremor score of 9 tremor items (right and left arm tremor by history, lips or chin tremor, tremor in all 4 limbs, and both arms action or postural tremor on examination) as well as a mean score of 5 PIGD items (i.e., falling, freezing, and walking difficulty by history, gait and postural instability on examination). Patients were assigned to the tremor group (TD) if the ratio of the mean tremor score divided by the mean PIGD score was greater than or equal to 1.5. Patients were assigned to the PIGD group if this ratio was equal to or less than 1.0. When the tremor/PIGD ratio was more than 1.0 and less than 1.5, patients were classified as undetermined. In addition, in order to assess correlations between motor symptoms and WMHs, we used a total TD and PIGD score rather than the ratio (i.e., the sum of 5 items for the PIGD score, and the 9 items for the tremor score).

Parkinsonian symptoms and disease severity were measured using the MDS-UPDRS introduced by Goetz et al. in 2008. The new Freezing of Gait questionnaire (FOG-Q) was used to assess if the subject experiences freezing episodes and its severity [12]; Subjects walked back and forth along a 35 meter corridor under single and dual task conditions (i.e., while subtracting serial 3s). Gait speed in the off state was determined by measuring the average time the subject walked along the middle 10 meters of a the corridor. The number of falls experienced one year prior to participation in the study was collected via self-report. The cognitive assessment included the Montreal Cognitive Assessment (MoCA) and the Mini Mental State Exam (MMSE). We also assessed depressive symptoms using the Geriatric Depression Scale.

*MRI acquisition*: A high-resolution T1-weighted brain volume (BRAVO) acquisition was used with the following parameters to ascertain Gray Matter (GM) changes: Repetition Time (TR) = 9000 milliseconds, TE = 3.6 milliseconds, flip angle = 90, voxel size = 1×1x1, matrix = 256x256, FOV = 250X250 mm2.

The resting-state fMRI scans were performed using an echo planar imaging (EPI) sequence with the following scan parameters: Repetition Time (TR) = 1,680 milliseconds, TE = 35 milliseconds, flip angle = 90, slice thickness = 3.5 mm, matrix = 64x64, FOV = 200x200 mm2.

The raw dataset provided by the Tel Aviv Sourasky Medical Center consists of demographics, clinical and neuroimaging information collected from 105 patients with PD. We removed several irrelevant variables that were not used in the first analysis. There were 66 missing values including mislabeled cells. Two subjects were excluded during preprocessing due to the missingness in the outcome variable of interest: *Fall_1_year (i.e., number of falls in the past year)*. Since missing values were only observed in the numerical variables, data imputation using chained equations was carried out based on Predictive Mean Matching (PMM) [13]. Median of the 5 imputed values were used to replace the missing cells. From the derived neuroimaging biomarkers, the *background* volume was removed because it represents extra-cerebral volume and it is not related to the clinical outcome. Similarly, TD and PIGD classification as well as Tremor score and PIGD score are obtained (equivalent to MDS_TREM and MDS_PIGD). Again, all numerical variables were centered around the mean and scaled by their standard deviation. The numerical variable *Fall_1_year* originally presented the number of falls recorded for each participant in the past year. In our study, we binarized the fall outcome variable to represent an indicator of patients’ history of falls (0 if patient had 0 fall vs. 1 if patient had one or more falls). Eventually, the processed Tel-Aviv dataset included 103 observations of 165 variables.

### **I.2 Predictive Analytics**

**I.2.a Model-based learning method**

***Logistic regression*:** Logistic regression was developed in 1958 by David Cox [14] for problems where the dependent variable is categorical. The probability of a response can be estimated through a linear model. Binomial logistic regression is employed when the response is dichotomous, in this study “Fall” vs. “No Fall”. If there are more than two categories in the response, multinomial logistic regression is useful. To sum up; we have a binary output variable $Y$ and we want to model the conditional probability $\Pr\left( Y=1 | X=x \right)=p(x)$ as a function of $x$. Formally, the logistic regression model is that:

$log\frac{p(x)}{1-P(x)}=\beta_{0}+x\cdot\beta.$ (1)

Solving for $p\left( x \right)$ yields:

$p\left( x;\beta_{0}, \beta\right)= \frac{e^{{\{\beta}_{0}+x\cdot\beta)}}{1+e^{{\{\beta}_{0}+x\cdot\beta)}}= \frac{1}{1+e^{-{\{\beta}_{0}+x\cdot\beta)}}.$ (2)

### **I.2.b Model-free learning methods**

***Random Forest*:** Random Forest [15] is an ensemble machine learning method for classification and regression. The logic behind the ensemble method is that a group of “weak learners” are combined and form a “strong learner”. We used the R package *Caret* [16] with 5-fold cross validation for model training and prediction. In the training process, Random Forest grows a number of decision trees (i.e. “weak learner”) with subsets of the original data (created by bootstrap) and randomly selected variables. When the input enters the system, it goes through all the trees and the result is the average, weighted average or majority vote of outcomes at all terminal nodes. With the idea of bootstrap aggregation (“bagging”), random forest is able to achieve stable prediction by decreasing variance and avoid overfitting. The algorithm includes the following three steps:

- Step1. Create $B$ bootstrapped (i.e. sampled with replacement) training data sets. The observations not sampled are called an out-of-bag sample (OOB) (one is automatically created for each bootstrap sample);
- Step2 Grow a decision tree on each bootstrap sample, considering $m$ randomly selected variables for each split. Recommended values of m range from 1, 2 to $log(p)$ or $\sqrt{p}$, generally much less than $p$;
- Step3 Use simple majority vote of $T_{1}\left( x \right),\ldots,T_{B}(x)$ to classify a new observation $X$.

***Adaboost*:** short for “Adaptive Boosting” [17] is another example of ensemble meta-algorithm method, which is able to improve prediction performance by primarily reducing bias. Similar to bagging, boosting converts weak learners into a strong one [18]. AdaBoost is the first realization of boosting in application with great success. It is best at boosting the performance of binary classification with decision trees. In the training process, equal weight is assigned to the sampled training instances when the first weak model is fitted. Then this method is adaptive in the sense that subsequent weak models are modified in favor of incorrectly predicted instance by giving them more training weight. For each weak model, a stage value is calculated using misclassification rate, which acts as a weight for prediction that model makes. The weak models trained on the weighted training data are added sequentially with stage values to form the strong learner. The iterative process stops when strong learner reaches the pre-set maximum number of weak learners or optimized performance. AdaBoost and related algorithms were categorized as ARCing (Adaptive Reweighting and Combining) algorithms in a statistical framework [19].

Due to its learning mechanism, AdaBoost in general is sensitive to outliers and noisy data which potentially leads to overfitting. The algorithm includes the following steps:

Initialize the observation weights $W_{i}=\frac{1}{n}, i=1,2,\ldots,n$. For $M=1,\ldots,M$, repeat:

- Step 1. Fit a classifier $T_{m(x)}$ to the training data using weights $\alpha_{m}=log(\frac{1-{err}_{m}}{{err}_{m}})$;
- Step 2. Compute its classification error on the training data, ${err}_{m}=\sum_{i=1}^{n} W_{i}I(y_{i}\neq T_{m}(x_{i}))$;
- Step 3. Compute classifier weight $\alpha_{m}=log(\frac{1-{err}_{m}}{{err}_{m}})$;
- Step 4. Update weights for $i=1,\ldots,n$: $w_{i}\leftarrow w_{i}\cdot e^{\alpha_{m\cdot I(y_{i}\neq T_{m}\left( x_{i} \right))}}$ and renormalize $w_{i}$ to sum to 1.

The final classifier is a weighted majority vote, $C\left( x \right)=sign(\sum_{m=1}^{M} T_{m}(x))$.

***XGBoost(Gradient Boosting)****:* XGBoost stands for “Extreme Gradient Boosting”, which is an implementation of gradient boosting machines created by Tianqi Chen in 2014 [20]. Similar to other boosting methods like AdaBoost, Gradient Boosting, developed by Friedman, produces a prediction model via an ensemble of weak leaners (decision trees) in a stage-wise fashion for solving regression and classification problems. As a generalization of boosting, Gradient Boosting works as a numerical optimization problem. Given an arbitrary differentiable loss function, the goal is to minimize the loss of the model by adding weak learners like a gradient descent algorithm. Hence, it allows the technique to deal with regression and multi-class classification besides binary classification problems. XGBoost is a popular library focused on computational speed and model performance. XGBoost allows Parallelization, Distributed Computing, Cache Optimization, etc. and supports Gradient Boosting, Stochastic Gradient Boosting and Regularized Gradient Boosting. The implementation of algorithm is carefully engineered for best efficacy. XGBoost is one of the top choice of winners for classification and regression predictive modeling challenges on many competitive data science platforms like Kaggle.

For a given data set with $n$ observations and $m$ features, $D=\{(x_{i},y_{i})\}(x_{i}\in R^{m})$, a tree ensemble model uses $K$ additive functions to predict the output.

$\hat{y_{i}}=\phi\left( x_{i} \right)=\sum_{k=1}^{K} f_{k}(x_{i}), f_{k}\in F,$ (3)

where $F=\left\{ f\left( x \right)=w_{q\left( x \right)} \right\}\left( q:R^{m}\to T,w\in R^{T} \right)$ is the base function space. Here $q$ represents the structure of each tree that maps an observation to the corresponding leaf index. $T$ is the number of leaves in the tree. Each $f_{k}$corresponds to an independent tree structure $q$ and leaf weights $w$. To learn the set of functions used in the model, we minimize the following regularized objective:

$L\left( \phi\right)=\sum_{i} l\left( \hat{y}_{i},y_{i} \right)+\sum_{i} \Omega\left( f_{k} \right), where \Omega\left( f_{k} \right)= \gamma T+\frac{1}{2}\lambda$. (4)

Here $l$ is a differentiable convex loss function that measures the difference between the prediction $\hat{y}_{i}$and the target $y_{i}$. The second term $\Omega$ penalizes the complexity of the model. The additional regularization term helps to smooth the final learnt weights to avoid overfitting. When the regularization parameter λ is set to zero, the objective falls back to the traditional gradient tree boosting.

However, the tree ensemble model includes functions as parameters and cannot be optimized using traditional optimization methods in Euclidean space. Instead, the model is trained in an additive manner. Besides the regularized objective, two additional techniques are used to further prevent overfitting. The first technique is shrinkage. Shrinkage scales newly added weights by a factor $\eta$ after each step of tree boosting. Similar to a learning rate in stochastic optimization, shrinkage reduces the influence of each individual tree and leaves space for future trees to improve the model. The second technique is column subsampling. This technique is also used in Random Forest. According to user feedback, using column subsampling prevents overfitting even more so than the traditional row subsampling.

***Support Vector Machines*:** Support Vector Machines (SVMs) are supervised learning methods [21] for classification and regression. In SVM, $n$ numeric covariates (categorical input needs to be converted into dummy variables) in the dataset form an n-dimensional space and a hyperplane (linear classifier) is selected for separation of points from different class, assuming the data are linearly separable. The distance between the hyperplane and the nearest data points on each side is called the margin. The hyperplane with the largest margin is preferred and referred to as Maximal-Margin hyperplane (Maximal-Margin Classifier). Those closest points are the support vectors as they define the hyperplane. However, the data in real world practice usually cannot be separated by a linear classifier in that space. Hence, the soft margin classifier is introduced, which allows some data points to violate the separating plane. The tuning parameter $C$ defines the amount of violation allowed across all dimensions. In this case, the support vectors are the instances within the margin. Further, it was proposed to map finite-dimensional space into a space with much higher dimensions for better separation. The SVM algorithm is implemented by application of kernel functions, such as Polynomial Kernel and Radial Kernel. In prediction, the kernel defines a distance measure between new input and the support vectors. In linear SVM, the dot product is the measure of similarity as the distance is a linear combination of the inputs, which can be replaced by kernel function.

Mathematically, optimize objective function:

$\min\omega\left( \alpha\right)=\frac{1}{2}\sum_{i=1}^{m} \sum_{j=1}^{m} y_{i}y_{j}\alpha_{i}\alpha_{j}\cdot K\left( x_{i},x_{j} \right)-\frac{C}{2}\sum_{j=1}^{m} \alpha_{j}$ (4)

$s.t. \sum_{i=1}^{m} y_{i}\cdot\alpha_{i}=0, 0\leq\alpha_{i}\leq c$,

where $K\left( x,z \right)=\left\langle\phi\left( x \right),\phi\left( z \right) \right\rangle$ is a kernel function.

The Lagrangian is solved by Dual problem under the Karush-Kuhn-Tucker (KKT) condition. Denote the optimal Lagrange multipliers as $\alpha^{*}=\left( \alpha_{1}^{*}, \alpha_{2}^{*}, \ldots, \alpha_{m}^{*} \right)^{T}.$ Then, the optimal solution is:

$b^{*}=y_{j}-\sum_{i=1}^{m} y_{i}\alpha_{i}^{*}K\left( x_{i}, x_{j} \right)$ (5)

$f\left( x \right)=sgn\left( \sum_{i=1}^{m} y_{i}\alpha_{i}^{*}K\left( x_{i}, x_{j} \right)+b^{*} \right)$. (6)

***Artificial Neural Network*:** The Artificial Neural Networks (ANNs) are computational models (algorithms) that mimic the biological neuronal structure of human brain on a smaller scale [22]. The basic unit for computation in ANN is the neuron, which is commonly referred to as a node. ANNs may have hundreds or thousands of nodes whereas there are approximately 100 billion neurons in a human brain. In a single neuron, when the inputs with assigned weights are fed, the node applies proper activation functions to the weighted sum of inputs to calculate the output. The simplest version of ANNs is Feedforward Neural Network, in which the information only moves forward. One example is multi-layer perceptron (MLP). It consists of multiple nodes arranged in layers: input layer, hidden layer(s) and output layer. The connections between nodes of adjacent layers have associated weights. Assigning correct weights for these connections is the ultimate goal of learning. In this study, the MLP learns through Stochastic Gradient Descent by the Back-Propagation algorithm.

Mathematically, let’s assume that the neural network has $L$ layers, **Supplementary Figure 1**. $x_{0}$ is the input vector, $x_{L}$ is the output vector and $t$ is the truth vector. The weight matrices are $W_{1}, W_{2}, \ldots, W_{L}$ and activation functions are $f_{1}, f_{2}, \ldots, f_{L}$. The optimization iteratively consist of three procedures:

Forward Pass:

$x_{i}=f_{i}(W_{i}x_{i-1})$ (7)

$E={\parallel x_{L}-t\parallel}_{2}^{2}.$ (8)

Backward Pass:

$\delta_{L}=(x_{L}-t)\circ f_{L}^{'}(W_{L}x_{L-1})$ (9)

$\delta_{i}=W_{i+1}^{T}\delta_{i+1}\circ f_{i}^{'}\left( W_{i}x_{i-1} \right).$ (10)

Weight Update:

$\frac{\partial E}{\partial W_{i}}=\delta_{i}x_{i-1}^{T}$ (11)

$W_{i}=W_{i}-\alpha W_{i}\circ\frac{\partial E}{\partial W_{i}}.$ (12)


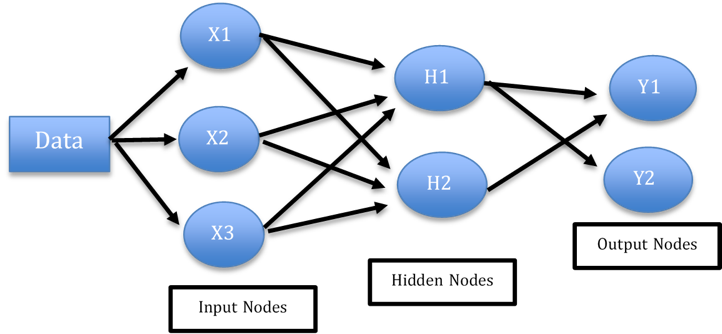


**Supplementary Figure 1**: Topology of Neural Network, arrow direction is a forward iteration. $X_{1}$, $X_{2}$ and $X_{3}$ are Input nodes, which should be equal to the number of the Input features. Multiple Hidden Nodes can be following. The output nodes correspond to the number of the labels of output.

***SuperLearner*:** Super learner is a loss based learning method that has been proposed and analyzed theoretically in van der Laan [23]. SuperLearner is a prediction method designed to find the optimal combination of a collection of prediction algorithms including both regression and classification. The super learner algorithm finds the weight for each algorithms, which minimize the cross-validated risk. Due to the previously established results, the super learner has been proven to outperform any of the given candidate estimators. For a given problem, a library of prediction algorithms can be included. The algorithms in the library may range from a simple linear regression to a complex algorithm like XGBoost. Super learner can also work as tuning parameters in a specific algorithm. From the oracle results established in previous articles, SuperLearner performs asymptotically as well as best possible weighted combination.

Observe the learning data and set $X_{i}=\left( Y_{i},W_{i} \right), i=1,\ldots,n$, where $Y$ is the outcome of interest and $W$ is a p dimensional set of covariates. The objective is to estimate function: $\phi_{0}\left( W \right)=E(Y|W)$. The function can be expressed as the minimizer of the expected loss:

$\phi_{0}\left( W \right)=\arg{min}_{\phi}E[L(X,\phi(w)]$, (13)

where the loss function is often the squared error loss: $L_{2}:{(Y-\phi(w))}^{2}$. For a given problem, a library of prediction algorithms can be proposed. A library is simply a collection of algorithms. The algorithms in the library should come from contextual knowledge and a large set of default algorithms. The algorithms may range from a simple linear regression model to a multi-step algorithm involving screening covariates, optimizing tuning parameters, and selecting a working model among a large class of candidate working models. As long as the algorithm takes the observed data and outputs a predicted value, we consider it a prediction algorithm.

### **I.3 Feature Selection**

**Method1: Random Forests**

N=500 iterations in total. In each iteration, 63.2% subjects are included. Variable importance is calculated based on the comparison of MSE before and after permutation. 20 top-ranked features (based on %IncMSE) are selected and the result for every iteration could be different. Hence, for feature$i$ we enumerate the number of times being in top 20, denoted $n_{i}$ and obtain its probability of being selected $p_{i}=n_{i}/N$. High $p_{i}$ indicates feature $i$ is critical for prediction.

**Algorithm for %IncMSE calculation**:

(1) Compute mean-square-error (MSE) for current model;

(2) For each ith feature: permute feature i and compute MSE_j for the new model;

(3) Calculate percentage increase in MSE (%IncMSE).

Generally, larger %IncMSE indicates higher importance.

**Method2: Knockoff Filtering**

N=1000 iterations in total. In each iteration, 63.2% subjects are included. We randomly choose 40 features as we want to the matrix at a lower dimension. The *knockoff.filter()* method in the knockoff R package is utilized to select promising features (FDR=0.35). Similarly, the number of times of survival in feature selection($n_{i}$) is recorded. The probability of being selected is adjusted for variable sampling ratio $\left( p_{i}=\frac{n_{i}}{N}\times\frac{\#features}{40} \right)$.

**Algorithm for knockoff filtering**:

(1) Construct null features of original covariates;

(2) Calculate appropriate statistics (Lasso in this study) for original and knockoff feature pairs;

(3) Set a threshold for the statistics with a target FDR and features are selected.

### II. Auxiliary Results

The figures and tables included in this auxiliary results section provide supporting information for the findings reported in the main article.

**Supplementary Figure S.2** shows for each dataset surface plots of sets of relations of three features (MOT_EDL, gaitSpeed (Off state) and MDS_PIGD) illustrating non-linear association patterns.


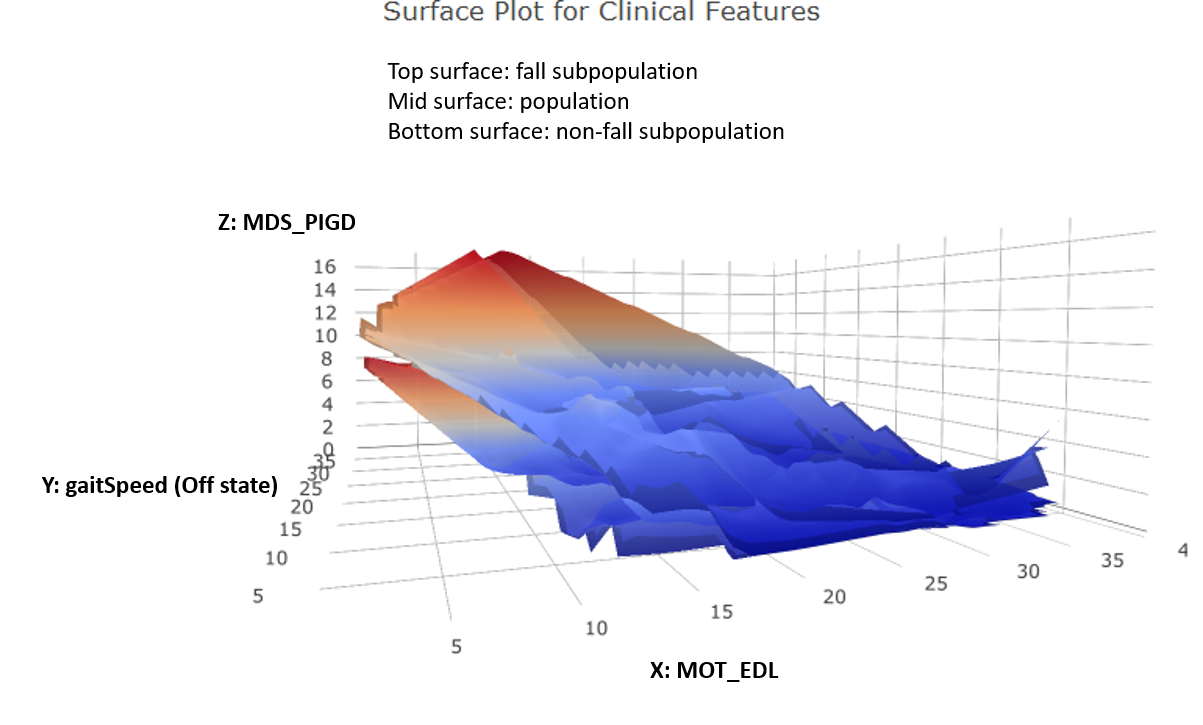


**Figure S.2.a**: (Michigan) MOT_EDL, gaitSpeed (Off state) and MDS_PIGD scores are important clinical features related to Parkinson ’s disease. The surface plots exposes differences between the distributions of MOT_EDL, gaitSpeed (Off state) and MDS_PIGD scores between fall and non-fall patient population.

**
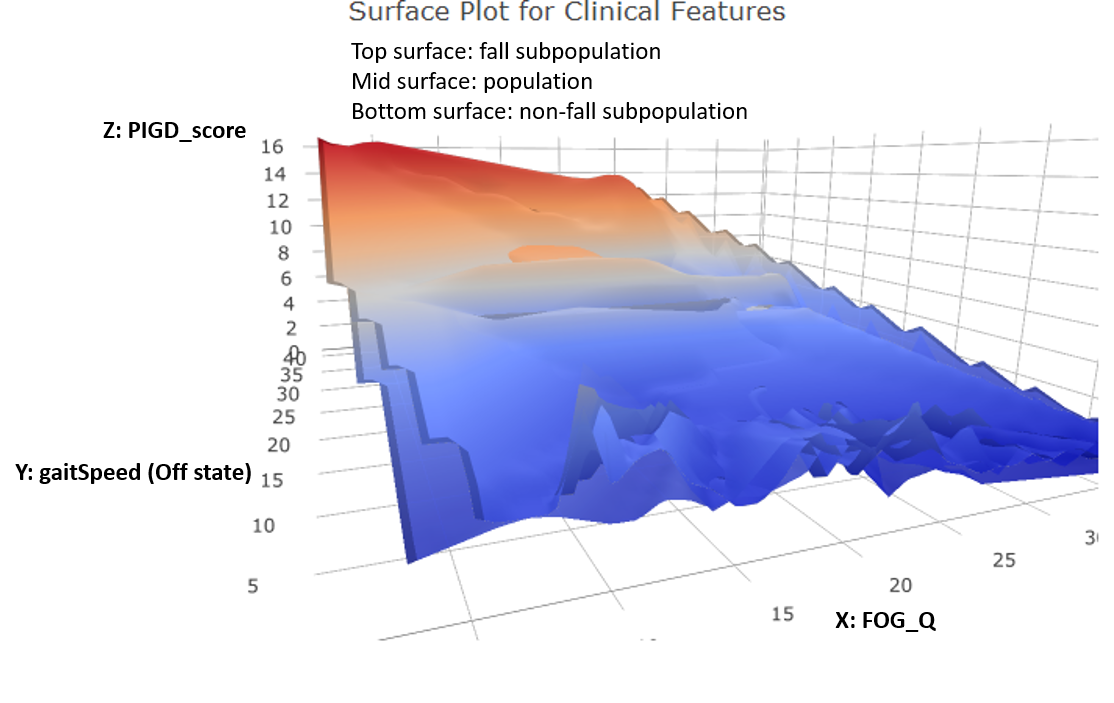
**

**Figure S.2.b**: (Tel-Aviv) PIGD, Tremor and FOG_Q scores are important clinical features related to Parkinson ’s disease. The surface plots expose differences between the distributions of PIGD, Tremor and FOG_Q scores between fall and non-fall patient population.

**Supplementary Figure S.3** depicts more elaborate feature-pair plots for each archive.


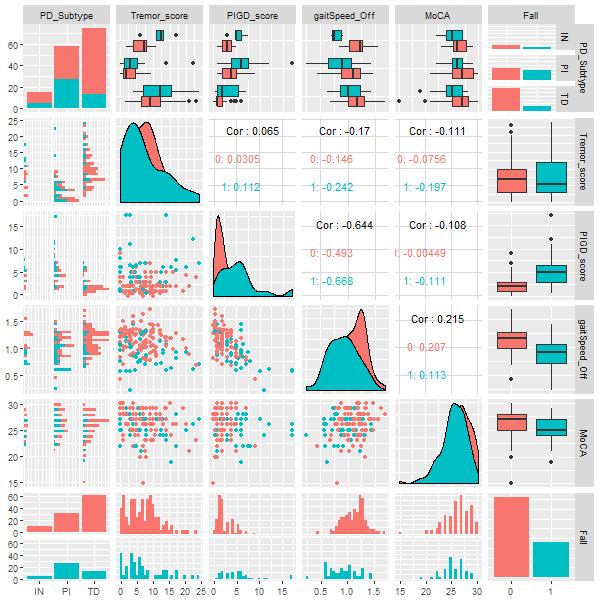


**Figure S.3.a**: Pairs plot for selected clinically important features in the Michigan dataset.


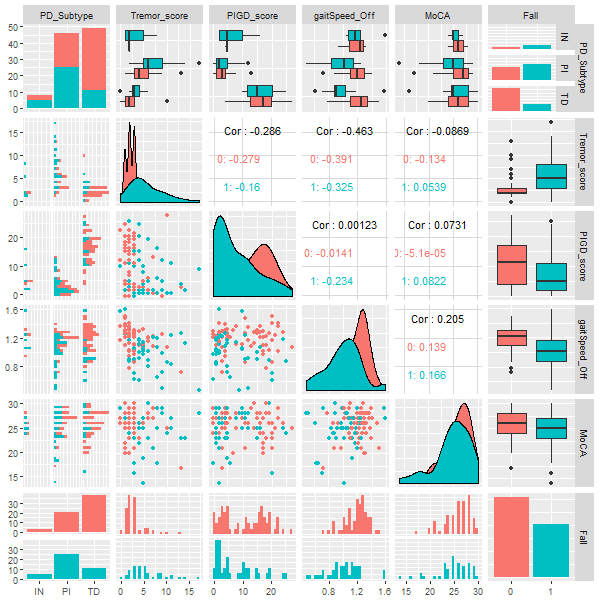


**Figure S.3.b**: Pairs plot for selected important features in the Tel-Aviv dataset.

**Supplementary Figure S.4** shows the results of multidimensional scaling projection of the original data points on the first two MDS dimensions, suggesting that the Falls/No-fall classification is challenging (Tel-Aviv data).


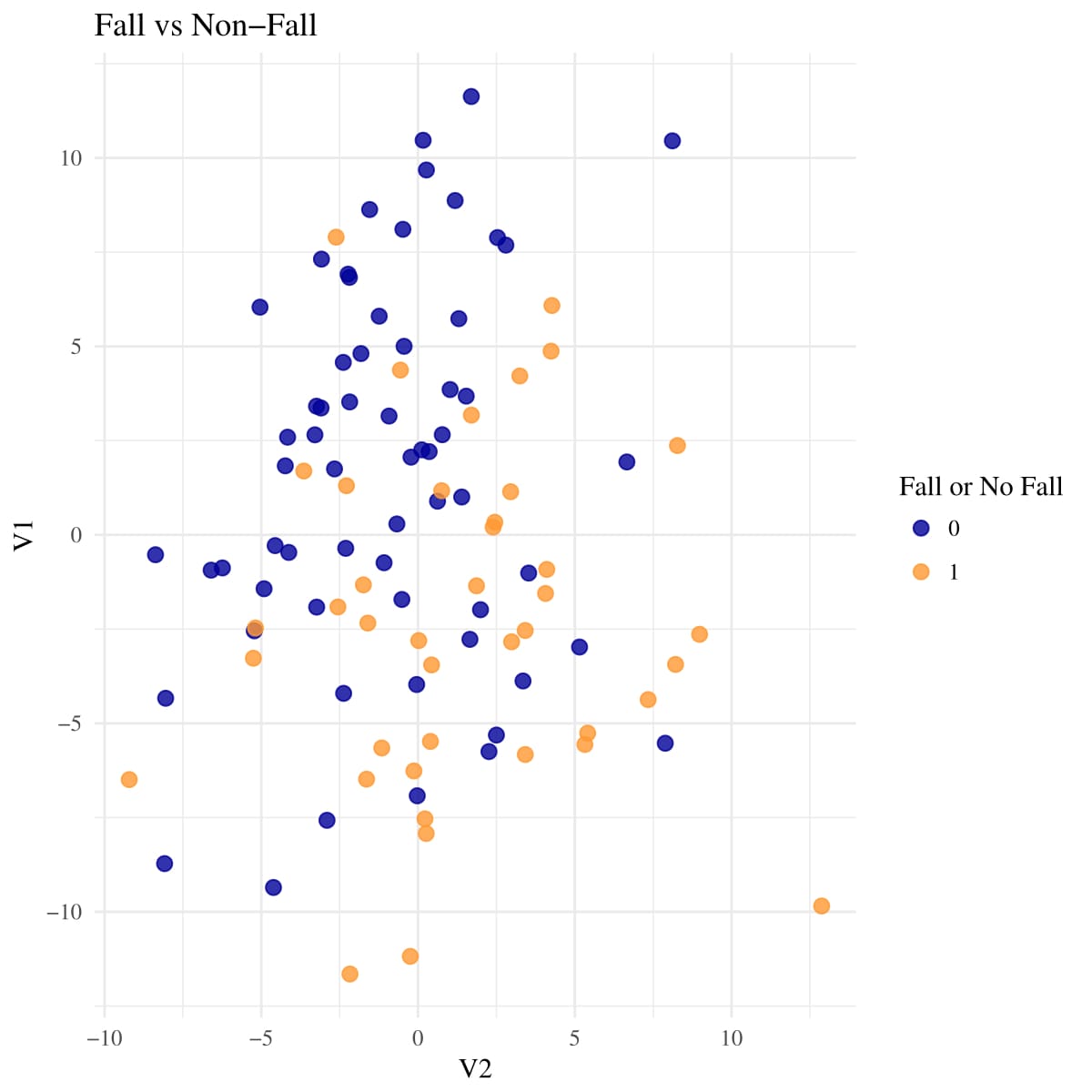


**Figure S.4**: Results of multidimensional scaling (MDS) projection of the original data points on the first two MDS dimensions, suggesting that the Falls/No-fall classification is challenging (Tel-Aviv data).

**Supplementary Figure S.5** shows the projection of the unsupervised learning for Tel-Aviv data on 2 dimensions MDS coordinates.


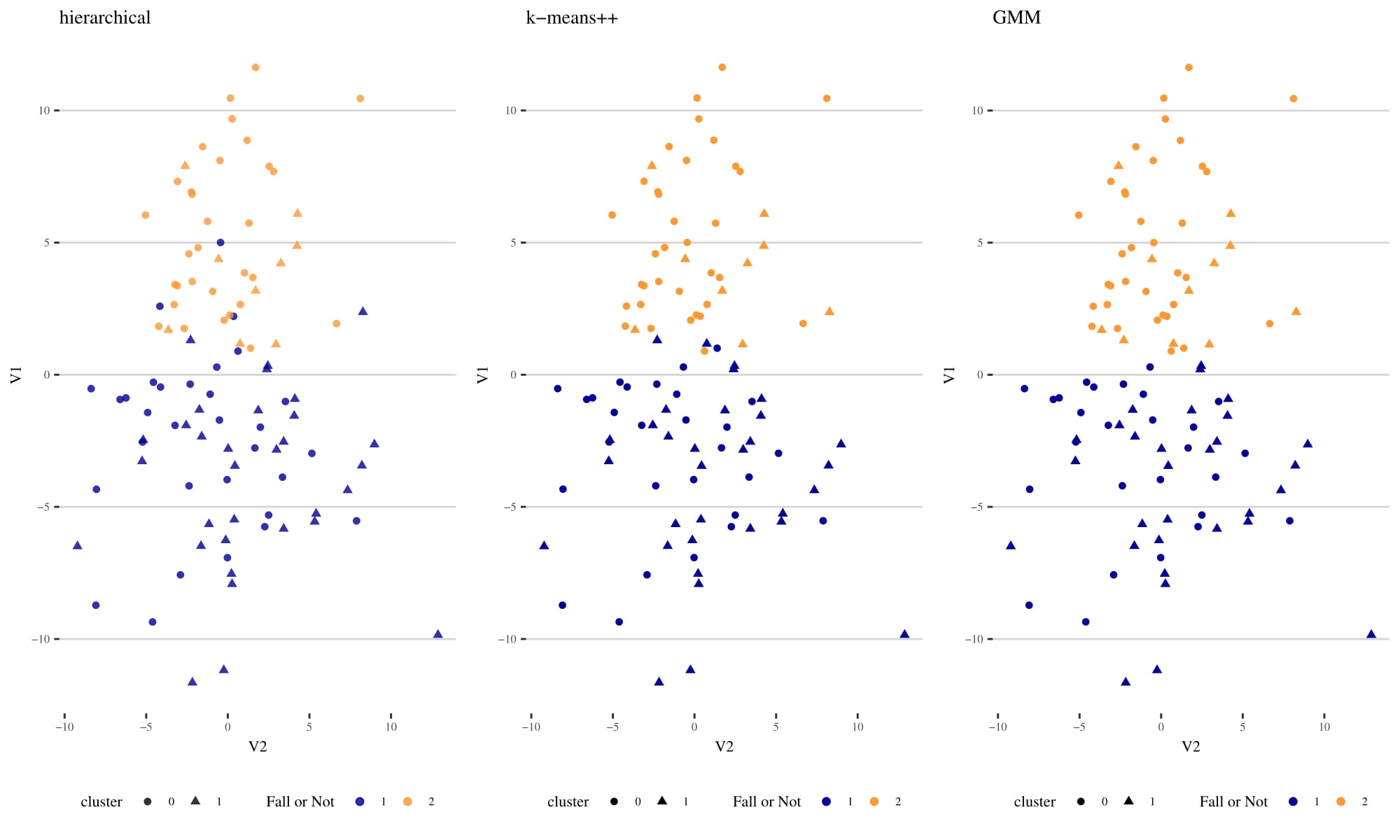


**Supplementary Figure S.5**: Unsupervised learning for Tel-Aviv data, projected on 2 dimensions MDS coordinates.

**Supplementary Table S.2**: Silhouette value of clustering for Tel Aviv data, projected on 2 dimensions MDS coordinates.

| Method | Mean Silhouette | Median Silhouette | Silhouette Range |
| --- | --- | --- | --- |
| Hierarchical clustering | 0.071 | 0.079 | (-0.158,0.261) |
| Kmeans++ | 0.092 | 0.095 | (-0.067,0.259) |
| Gaussian Mixture model(GMM) | 0.092 | 0.089 | (-0.066,0.259) |

**Supplementary Table S.3**: Results of comparing clinical feature classification accuracy against a latent variable model.

| Method | acc | sens | spec | ppv | npv | lor |
| --- | --- | --- | --- | --- | --- | --- |
| 5 Clinical feature | 0.689 | 0.561 | 0.774 | 0.622 | 0.727 | 1.477 |
| Clinical features Latent model | 0.680 | 0.561 | 0.758 | 0.605 | 0.723 | 1.387 |

To predict falls of patients with only 5 clinical features, a latent variable model was fitted for the continuous clinical features: PIGD Score, Tremor Score. The coefficient of these three variables were estimated to be 1.0 and -0.563 respectively. The classification performance using the top 5 clinical features (PIGD_score, Tremor_score, FOG_Q, H_and_Y_OFF, gaitSpeed_Off) and the latent variable are shown on Supplementary **Table S.3**.

# References

1. Hughes, A.J., et al., *Accuracy of clinical diagnosis of idiopathic Parkinson's disease: a clinico-pathological study of 100 cases.* J Neurol Neurosurg Psychiatry, 1992. **55**(3): p. 181-4.

2. Hoehn, M. and M. Yahr, *Parkinsonism: onset, progression, and mortality.* Neurology, 1967. **17**: p. 427-442.

3. Chawla, N.V., Bowyer, Kevin W., Hall, Lawrence O., Kegelmeyer, W. Philip, *SMOTE: synthetic minority over-sampling technique.* Journal of artificial intelligence research, 2002: p. 321-357.

4. Buuren, S. and K. Groothuis-Oudshoorn, *MICE: Multivariate imputation by chained equations in R.* Journal of Statistical Software, 2011. **45**(3).

5. Stebbins, G.T., et al., *How to identify tremor dominant and postural instability/gait difficulty groups with the movement disorder society unified Parkinson's disease rating scale: comparison with the unified Parkinson's disease rating scale.* Movement Disorders, 2013. **28**(5): p. 668-670.

6. Rosenberg-Katz, K., et al., *Subcortical Volumes Differ in Parkinson’s Disease Motor Subtypes: New Insights into the Pathophysiology of Disparate Symptoms.* Frontiers in human neuroscience, 2016. **10**.

7. Bernad-Elazari, H., et al., *Objective characterization of daily living transitions in patients with Parkinson’s disease using a single body-fixed sensor.* Journal of neurology, 2016. **263**(8): p. 1544-1551.

8. Weiss, A., et al., *Association between community ambulation walking patterns and cognitive function in patients with Parkinson’s disease: further insights into motor-cognitive links.* Parkinson’s Disease, 2015. **2015**.

9. Weiss, A., et al., *Does the evaluation of gait quality during daily life provide insight into fall risk? A novel approach using 3-day accelerometer recordings.* Neurorehabilitation and neural repair, 2013. **27**(8): p. 742-752.

10. Arie, L., et al., *Do cognition and other non-motor symptoms decline similarly among patients with Parkinson’s disease motor subtypes? Findings from a 5-year prospective study.* Journal of neurology, 2017. **264**(10): p. 2149-2157.

11. Jankovic, J., K. Schwartz, and D.T. Donovan, *Botulinum toxin treatment of cranial-cervical dystonia, spasmodic dysphonia, other focal dystonias and hemifacial spasm.* Journal of Neurology, Neurosurgery & Psychiatry, 1990. **53**(8): p. 633-639.

12. Nieuwboer, A., et al., *Reliability of the new freezing of gait questionnaire: agreement between patients with Parkinson's disease and their carers.* Gait & posture, 2009. **30**(4): p. 459-463.

13. Landerman, L.R., K.C. Land, and C.F. Pieper, *An empirical evaluation of the predictive mean matching method for imputing missing values.* Sociological Methods & Research, 1997. **26**(1): p. 3-33.

14. Cox, D.R., *The regression analysis of binary sequences.* Journal of the Royal Statistical Society. Series B (Methodological), 1958: p. 215-242.

15. Breiman, L., *Random forests.* Machine learning, 2001. **45**(1): p. 5-32.

16. Kuhn, M., *Caret package.* Journal of Statistical Software, 2008. **28**(5).

17. Bauer, E. and R. Kohavi, *An empirical comparison of voting classification algorithms: Bagging, boosting, and variants.* Machine learning, 1999. **36**(1-2): p. 105-139.

18. Breiman, L., *Arcing classifier (with discussion and a rejoinder by the author).* Ann. Statist., 1998. **26**(3): p. 801-849.

19. Breiman, L., *Prediction games and arcing algorithms.* Neural computation, 1999. **11**(7): p. 1493-1517.

20. Chen, T. and C. Guestrin. *Xgboost: A scalable tree boosting system*. in *Proceedings of the 22nd acm sigkdd international conference on knowledge discovery and data mining*. 2016. ACM.

21. Hearst, M.A., Dumais, Susan T, Osman, Edgar, Platt, John, Scholkopf, Bernhard, *Support vector machines.* Intelligent Systems and their Applications, IEEE, 1998. **13**(4): p. 18-28.

22. Bishop, C.M., *Neural networks for pattern recognition*. 1995: Oxford university press.

23. Van der Laan, M.J., E.C. Polley, and A.E. Hubbard, *Super learner.* Statistical applications in genetics and molecular biology, 2007. **6**(1).
